# Nests, Threats, and Leks: Nonrandom Distributions of Nests in Ruffs (*Calidris pugnax*)

**DOI:** 10.1002/ece3.70997

**Published:** 2025-02-28

**Authors:** Hanna Algora, James D. M. Tolliver, Veli‐Matti Pakanen, Krisztina Kupán, Jelena Belojević, Nelli Rönkä, Clemens Küpper, Kari Koivula

**Affiliations:** ^1^ Department of Ecology University of Oulu Oulu Finland; ^2^ Research Group Behavioural Genetics and Evolutionary Ecology Max Planck Institute for Biological Intelligence Seewiesen Germany

**Keywords:** flooding, habitat selection, nest‐site selection, predation, ruff, suitable breeding habitat, waders

## Abstract

Habitat selection determines an animal's spatial distribution at various scales. In ground‐breeding birds, selecting the right nesting location can be decisive for the survival of parents and offspring. However, it remains often unclear what cues birds use to settle in their breeding habitat. Ruffs (*Calidris pugnax*) are waders with highly divergent sex roles: males aggregate for competitive display to attract females (reeves), who then care for the nest and offspring alone. Ruffs frequently breed in coastal wetlands of higher latitudes where they often face the threat of nest loss because of flooding or predation. We investigated which environmental and social cues determine Ruff nest distributions in a coastal meadow habitat. Using nest locations from five breeding seasons and their relative distance to other nests, leks, the shoreline, and meadow edge, we tested whether Ruff nests are randomly distributed across the suitable breeding habitat or show some level of spatial association. We first compared average nearest neighbor (ANN) distances between Ruff nests (observed and simulated) and spatial features in univariate models. Then, we examined the effect of all spatial features on nest location in a multivariate generalized linear mixed model (GLMM) using a Bayesian framework. Our results show that nest distribution is nonrandom; nests of reeves are found closer to leks of male Ruffs and other nests than expected by chance. In some years, we found nests further away from the meadow edges and shore than expected by chance. Overall, our results suggest that nesting females may use social cues and the distance to habitat boundaries when choosing a nest site. We suggest that understanding the social and environmental factors affecting female nest choice can help to improve the management and conservation routines at the breeding sites of these threatened waders. Our results indicate that lekking sites may be used to identify nesting areas of conservation management value.

## Graphical abstract

This project investigates the nest distribution of the ruff (*Calidris pugnax*), an endangered shorebird. Using satellite imaging, we established a suitable breeding area and analyzed the factors influencing nest placement. Our study aims to provide insights that can aid the conservation of this species by understanding nesting behavior and outlining the threats nests face.

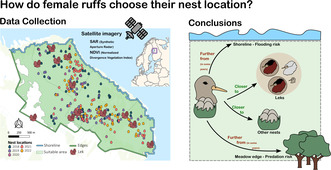

## 1 Introduction

Habitat selection largely determines the spatial distribution of animals. Selection refers to the way in which animals choose between patches, based on cues that ultimately affect their fitness (Boyce and McDonald 1999), leading to a disproportionate use of the habitat based on availability (Matthiopoulos et al. 2023). During decision‐making, animals utilize collective and individual experiences to gain information about the environmental properties that define a habitat and its quality (Ahlering and Faaborg 2006; Kristan et al. 2007). The trade‐off between maximizing resources such as access to food and mates, while minimizing predation threats and environmental hazards, is thought to lead to the observed patterns of population distribution and abundance (Boyce and McDonald 1999; Matthiopoulos et al. 2015; Northrup et al. 2022).

Habitat selection is of particular importance during reproduction. For birds, choosing an optimal nesting location can be decisive for the fitness of both adults and offspring; therefore, it bears immediate and long‐term consequences (Orians and Wittenberger 1991; Kristan et al. 2007). By using different environmental cues, birds may try to forecast relevant resources and threats, such as food availability (Blondel et al. 1993) and predator distributions (Söderström 2001; Kristan et al. 2007; Pass et al. 2019) prior to choosing their nest location. In some instances, however, birds fail to choose the optimal habitat. Opposing benefits between different habitat types, which require prioritization of certain fitness components over others, as well as unpredictability in the reliability of biotic and abiotic factors, might lead to suboptimal habitat choices (Kristan 2007; Kristan et al. 2007). In many bird species, predation has a major effect on reproductive fitness (Ricklefs 1969; Martin 1993a, 1993b; Suhonen et al. 1994; McCollin 1998; McMahon et al. 2020; Kaasiku et al. 2022). Therefore, strong selection should operate on individuals to optimally choose nesting habitats and social environments, and evolve life‐history traits that minimize the negative effects of predation (Martin 1993b). Minimizing predation risk is particularly important for species breeding in environments where nest predation rates are high. Ground‐breeding waders that make open nests on coastal meadows and farmlands with limited visual cover, are one such group (Martin and Roper 1988; Seitz and Zegers 1993; Burke et al. 2004; Einarsen et al. 2008; Wegge et al. 2012; Pass et al. 2019). These species often prefer to place their nests in grassy areas, avoiding patches of high vegetation that could impede the mobility of their offspring (Devereux et al. 2004) or make it difficult for the parents to detect predators (Gómez‐Serrano and López‐López 2014).

Nest predation can be exacerbated by edge effects, which occur at the boundary between two abruptly different habitats (Sammalisto 1957; McCollin 1998; Huhta et al. 2015), such as transition areas between coastal meadows and forest habitats, by increasing the proximity of predators to nests and exposing nests to a higher abundance and diversity of predators. Edge effects can be especially strong for nests placed near habitat edges where many predator species prefer to travel (Andrén 1995; McCollin 1998). Therefore, nest distance from the habitat edges may be related to the relative risk of nest predation (Paton 1994; McCollin 1998), which tends to be stronger in areas close to the habitat edge (Rönkä and Koivula 1997; Hunter et al. 2016; Kaasiku et al. 2022). Despite the predicted lower risk provided by nesting in areas further from the inland meadow edges, birds may end up breeding closer to the shore and can be additionally threatened by increased flooding risk in environments where water levels vary (Erwin et al. 2006; Van de Pol et al. 2010; Hunter et al. 2016; Bailey et al. 2017, 2019; Koivula et al. 2025).

Social elements are an additional consideration for habitat choices. Some waders are characterized by high variation in sex roles, which include differences in competition for mates and parental care investment (Thomas et al. 2007; Liker et al. 2013). In biparental species where both parents provide care, habitat requirements should be similar for both sexes, as both are involved with care. However, when exclusively one parent provides care, the breeding habitat requirements for males and females can differ (Cody 1985; Devoucoux et al. 2019). Therefore, divergent sex roles can expand the disparity between the ideal breeding patch for males and females, generating sexually dimorphic habitat selection patterns (Morales et al. 2008; Devoucoux et al. 2019), with nest site selection being particularly relevant for the sex in charge of parental care (Cody 1985).

Lekking species may offer an illustrative example for divergent needs between the sexes in habitat requirements. Lekking males aggregate with the purpose of attracting females for mating but neither hold resources nor contribute to parental care (Beehler and Foster 1988; Höglund and Alatalo 1995). Males require sites of high visibility and proximity to food sources and females but also safety (Alonso et al. 2012; Devoucoux et al. 2019). Females require a safe and suitable space for nesting as they spend weeks on incubating the eggs. Leks may be indicators of suitable habitat and hence could play an important role in habitat selection for breeding females (Höglund and Alatalo 1995; Devoucoux et al. 2019). In some lekking species, such as the Little Bustard ( *Tetrax tetrax* ), females tend to nest close to the lekking grounds of males, though not in direct proximity of the leks in order to avoid harassment (Jiguet et al. 2002). In other species, females may choose to nest further away from areas of male display to minimize potential costs such as competition for food, harassment, and predator attraction (Wrangham 1980; Phillips 1990).

Ruffs (*Calidris pugnax*) provide a highly suitable study system to examine social and environmental determinants of the nest site selection process. Ruff breeding sites include coastal meadows that are often bordered by agricultural fields and forested areas. In this lekking species, females, which are called reeves, are solely responsible for nest site selection and provide uniparental care. When looking for an appropriate nest location, reeves may consider the distance to displaying males and threats such as the risks of predation and flooding. In addition, nests should not be too far from suitable feeding grounds that can be reached by the precocial chicks once they have hatched. In contrast, male Ruffs select open and elevated spots with good visibility to attract females, aiming to fulfill their primary goal of achieving copulations. The surroundings of these spots are not necessarily optimal in terms of avoiding nesting failures.

Nesting failure is one of the greatest threats for European wader populations (Macdonald and Bolton 2008; Laidlaw et al. 2015). Over the last decades, European wader populations breeding in low‐lying shore habitats have suffered from steep declines due to high predation rates (Macdonald and Bolton 2008, Laidlaw et al. 2015) and flooding risks (Cooley et al. 2022). The Ruff is no exception to this worrying trend and has become a species of conservation concern since their breeding populations are decreasing on a global scale (Zoeckler et al. 2003). The species has nearly disappeared from former breeding areas in Western Europe and is being classified as critically endangered in Finland due to a rapid decline in their breeding numbers (Hyvärinen et al. 2019). Given the conservation concern, understanding the habitat requirements of breeding females is essential for the implementation of informed species and habitat management.

Our study had two objectives to better characterize the nesting habitat choices of breeding reeves. First, we asked whether nests were randomly distributed across the available habitat or showed nonrandom patterns related to social and environmental spatial features using univariate analyses. Specifically, we examined whether nests were found closer to or further away from the leks, other nests, meadow edge, and shoreline than expected by chance. We hypothesized that female nest‐site choice and hence nest location may be affected by spatial features, with nests being closer to leks and other nests (social features) than expected by chance, while being further away from the shoreline and the meadow edges (environmental features). Second, after establishing that the nest distributions were nonrandom, we determined the relative importance of these social and environmental features in shaping nest distributions in Ruffs.

## 2 Methods

### 2.1 Study Site

We carried out our study at Pitkänokka meadows on the coast of the Bothnian Bay, Finland, in the Liminganlahti area (ca. 64.86° N, 25.27° E). Pitkänokka (ca. 600 ha) provides both migration stop‐over and breeding habitats for numerous endangered waders, including Southern Dunlins ( *Calidris alpina schinzii* ) and Ruffs (Pakanen et al. 2011). The site is surrounded by forests and agricultural lands. The coastal meadows have been managed through seasonally rotational subsidized cattle grazing as part of the EU agri‐environmental schemes (Pakanen et al. 2016), with cows grazing different sections of the meadow throughout the summer months.

The meadow habitat comprises a zonal structure caused by a land‐uplift generated through a glacial isostatic adjustment (Richter et al. 2012; Hünicke et al. 2015; Pellikka et al. 2018). Different habitat zones are parallel to the shoreline, starting with one dominated by low‐height grasses (with sedges, *Carex* sp., grasses such as *Festuca* sp. and *Calamagrostis* sp., and rushes such as *Juncus gerardii* ) ideal for ground‐nesting waders (Devereux et al. 2004; Durant et al. 2008; Chasov et al. 2019; Kaasiku et al. 2021). Taller plants such as reeds ( *Phragmites australis* ) replace the grasses with increasing distance from the shore, as reeds began to infiltrate and overgrow the grassy zones over the study period (Burnside et al. 2007; Kose et al. 2021; Kaasiku et al. 2021).

### 2.2 Leks and Nest Data

We studied breeding Ruffs at Pitkänokka over a 5‐year period, from 2018 to 2022. The field seasons started during the first week of May before lekking began, with the exact date depending on the start of the ice melt and continued until all the nests hatched at the end of July.

During the first 2 weeks of May, we mapped and monitored active leks. Males arrive for breeding before females and establish their lekking sites (Höglund and Alatalo 1995). We defined leks as locations where one or more individually identified males were displaying for at least 1 h on two or more consecutive days. Ruff males that hold territories during lekking, also known as residents, are often site‐faithful between and within years, which aided locating the main lekking areas. We used males' plumage characteristics to distinguish individual males as they are highly variable between, but fixed within, different individuals (Hogan‐Warburg 1966; Van Rhijn 1991; Widemo 1997; Vervoort and Kempenaers 2020). The first week after the residents occupied the known main leks, we regularly scouted the meadow for smaller or novel leks, walking through the field site in the first hours after sunrise, searching for locations with recurrently displaying males.

We started nest searches in the second half of May, after we had observed copulations during lek observations. We searched in teams of one to three people, either walking through the meadow to flush incubating reeves or rope‐dragging, with two people pulling a rope over the vegetation to flush birds from a short encounter distance. We used GPS tracks of fieldworkers to establish areas where search effort had been low. Later, we further surveyed low search effort areas, to ensure that the entire study area was covered by our nest searching effort. Once we found a nest, we took the GPS coordinates and marked the spot within a few meters of the nest using individual marks improvised from natural materials to minimize predator recognition. We counted, measured, and then floated the eggs to estimate the start of incubation (Liebezeit et al. 2007). To back‐calculate the initiation of the nest, the day when the first egg of a clutch was laid, we used a laying interval of the eggs of 1.25 days and an incubation period of 21 days (authors' unpublished observations, Giraldo‐Deck et al. 2022).

For our analyses, we excluded nests with missing information about the laying date or with locations that fell outside our delimited field site based on the noted coordinates.

### 2.3 Suitable Breeding Area

The assessment of Ruff nest distributions required the establishment of a suitable breeding area with adequate habitat for nesting. We used remote sensing data to establish suitable breeding areas for reeves within our field site, within and between years. As we did not conduct a site‐wide vegetation study to model and predict the available habitat, we calibrated the suitable vegetation areas using proxies for vegetation cover, the Normalized Difference Vegetation Index (NDVI) (Pettorelli et al. 2011) and Synthetic Aperture Radar (SAR) backscatter (Harmoney et al. 1997; Hadjimitsis et al. 2004; Kaasiku et al. 2021) estimated from satellite images.

We used the images from the Sentinel‐2 satellite (spatial resolution at 10 m) to establish NDVI values for our entire study area for the laying period, defined as the first to the last date of the egg laying period (Table 1, Table A1). We retrieved 167 images from the *Copernicus Scientific Data Hub* between the 14th of May and 25th of June 2018–2022 and cropped them to the area of Pitkänokka. We used the R toolbox *sen2r* to download and preprocess the imaging from Sentinel‐2 (Ranghetti et al. 2020). We computed the NDVI based on the Bottom‐of‐Atmosphere (*BOA*) products for reflectance of areas on the surface of the Earth, and then, we applied a *clear sky* cloud mask, allowing a maximum of 5% of masked area, leaving 41 NDVI images for further analysis. We could not retrieve any images for 2022 with a cloud coverage < 5%; hence, we did not include this year to establish a suitable breeding area based on NDVI values.

**TABLE 1:** Laying periods and number of nests and leks.

Year	Leks	Nests	Laying period
2018	6	64	16 May–24 June
2019	8	56	21 May–25 June
2020	4	28	20 May–15 June
2021	4	54	21 May–9 June
2022	6	44	14 May–23 June

*Note:* Nest and lek numbers for the years 2018–2022 indicating Ruff laying periods.

The SAR data were obtained from the Sentinel‐1 satellite and consist of a sensor that transmits and receives polarized microwaves, which can be used to infer land characteristics based on the backscatter of the returning waves (Hill et al. 1999). Unlike NDVI images, SAR enabled us to estimate the vegetation height of grassland meadows in all weather and visibility conditions (Hill et al. 1999; Kaasiku et al. 2021). We retrieved all sentinel images available for the laying interval (105 images between 15th May and 24th June, Tables A1 and A2; Copernicus Sentinel data 2018–2022, retrieved from ASF DAAC https://search.asf.alaska.edu, Alaska Satellite Facility, on 23 May 2023, processed by ESA, European Space Agency). We selected the subset of images from the satellite's orbit path 160 to minimize the impact of the satellite imaging angle on the backscatter caused by reflectance (Waite et al. 1981; Braun 2021), resulting in 18 images. We then preprocessed all images in batch using the ESA SNAP (Sentinel Application Platform; ESA 2022) software. Using the *Graph Builder*, we first subset the downloaded images to the extent of our field site. Then, we applied the default calibration and speckle filter on the subset and a terrain correction using the *Copernicus 30m Global DEM* (Digital Elevation Model) and converted the band data to decibels (dB).

**FIGURE A1:**
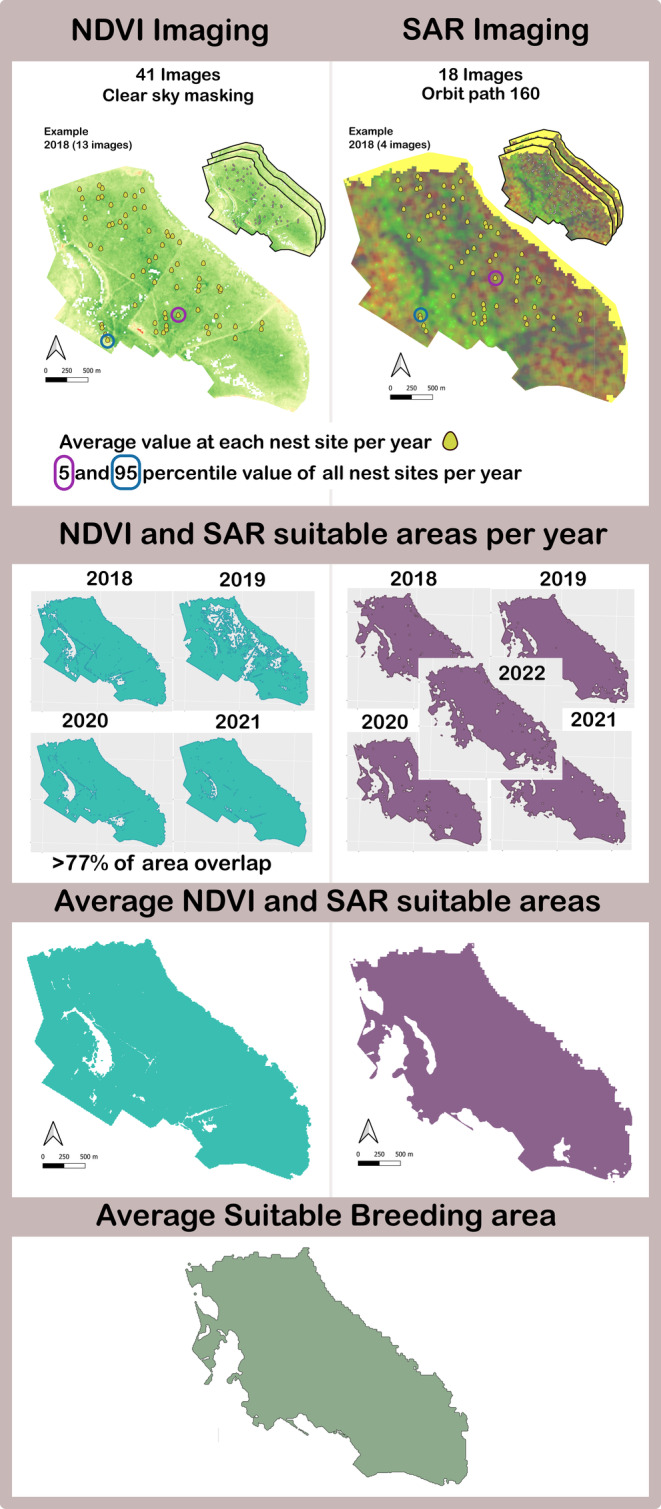
Suitable breeding area methodology. Schematic explaining the steps to generate the suitable breeding area.

**FIGURE A2:**
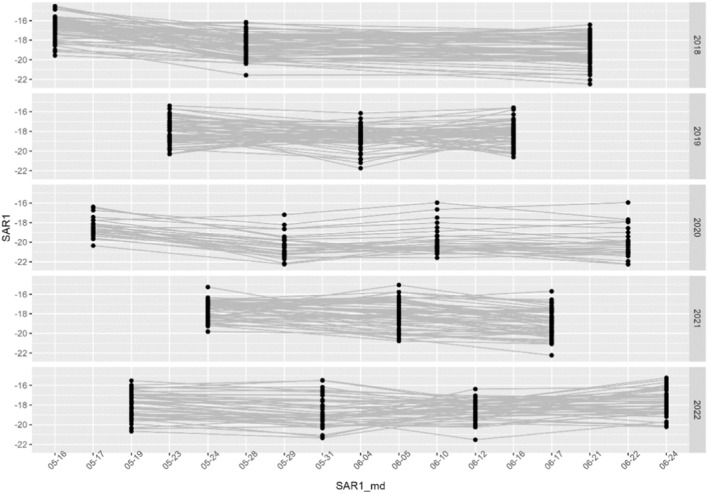
Within‐year SAR variation at nest locations. Within‐year SAR variation at nesting sites for the years 2018–2022. Gray line: Connects values of same nests.

**FIGURE A3:**
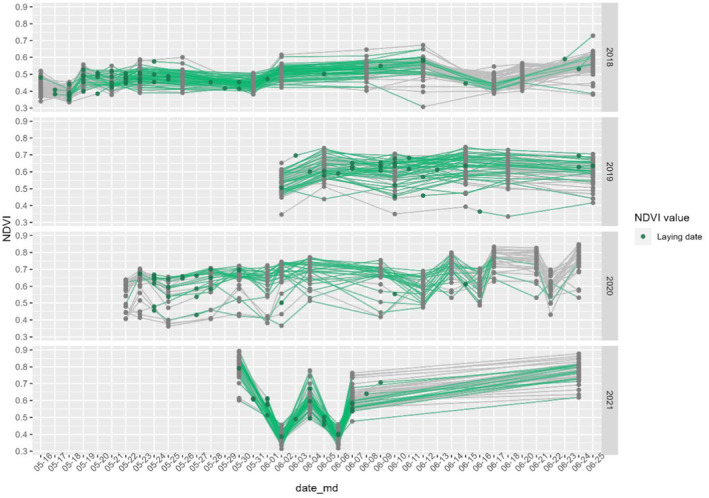
Within‐year NDVI variation at netst locations. Within‐year NDVI variation at nesting sites for the years 2018–2021 (no cloudless data available for the year 2022). Gray points: NDVI data point, Green points: NDVI at laying date, Gray line: Connects values of same nests, Green line: Nest active period.

We extracted the estimated NDVI and SAR values for each satellite reading at the exact nest locations for all the available dates (Figures A1, A2, A3). We then averaged these values per year to estimate the NDVI and backscatter for each nesting site accounting for possible outlier values coming from the satellite imaging. We used the 5 and 95 percentiles for the nest NDVI and SAR (Table 2) values to define the theoretical ranges at which Ruff nests could be laid, and we defined all pixels with values outside of these ranges as “unsuitable” (Figure A1). The average NDVI and SAR values at each nesting site based on the images available for each year ranged from 0.308 to 0.894 for greenness and from −22.48 to −14.52 dB for backscatter (Table 2).

**TABLE 2:** SAR (Synthetic Aperture Radar) and NDVI (Normalized Difference Vegetation Index) values at nesting sites.

Year	SAR *N*	Min dB	Max dB	NDVI *N*	Min NDVI	Max NDVI
2018	4	−22.48	−14.52	13	0.308	0.729
2019	3	−21.75	−15.38	6	0.335	0.747
2020	4	−22.26	−15.95	16	0.362	0.848
2021	3	−22.22	−15.06	6	0.314	0.894
2022	4	−21.54	−15.22	0	—	—
**Years**	**SAR *N***	**5% dB**	**95% dB**	**NDVI *N***	**5% NDVI**	**95% NDVI**
2018–2022	18	−20.96	−16.14	41	0.384	0.78

*Note:* Upper panel: Minimum and maximum average SAR and NDVI values for nesting locations for the years 2018–2022, indicating the number of images used. Lower panel: 5 and 95 percentile SAR and NDVI values for nesting locations for the years 2018–2022.

We assessed the vegetation cover using NDVI and SAR estimated from the satellite imagery data. The within‐year variation at nest sites was low in SAR (Figure A2), indicating little detectable changes in vegetation height specifically in Ruff breeding areas. The NDVI showed higher variation within years (Figure A3), reflecting the satellite's visibility conditions for a given year.

**FIGURE A4:**
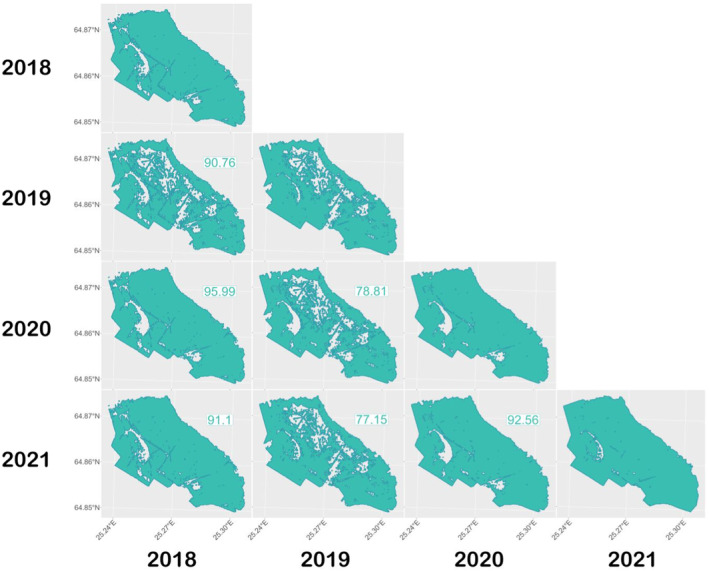
NDVI percentage area overlap. Intersection area and percentage of overlap between the suitable NDVI areas per year, from 2018 to 2021 (no cloudless data available for the year 2022).

**FIGURE A5:**
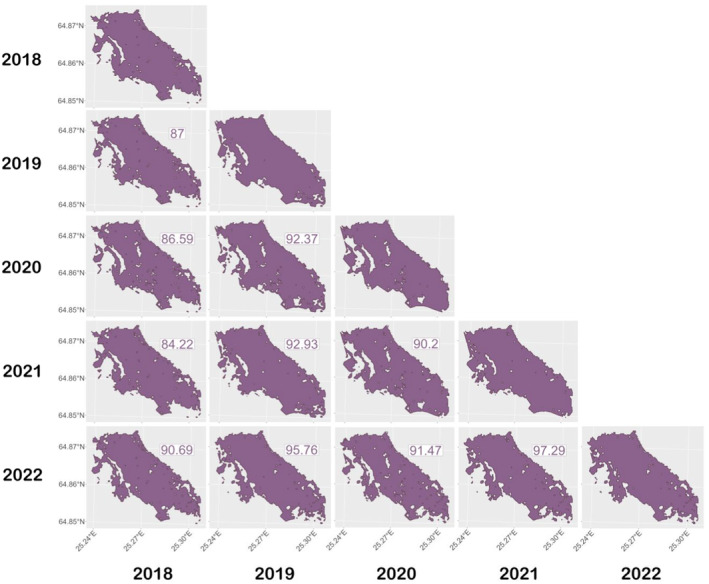
SAR percentage area overlap. Intersection area and percentage of overlap between the suitable SAR areas per year, from 2018 to 2022.

We compared the overlapping areas among all the years to assess the among‐year variation in the NDVI and SAR suitable areas. We determined that the NDVI and SAR suitable areas were similar between years (Figures A4 and A5), with a minimum overlap of more than 77%. Since the overlap of the yearly areas was considerable, as a final step, we averaged the theoretically available areas across all years, generating a single suitable NDVI and backscatter area, respectively (Figure A1).

**FIGURE 1:**
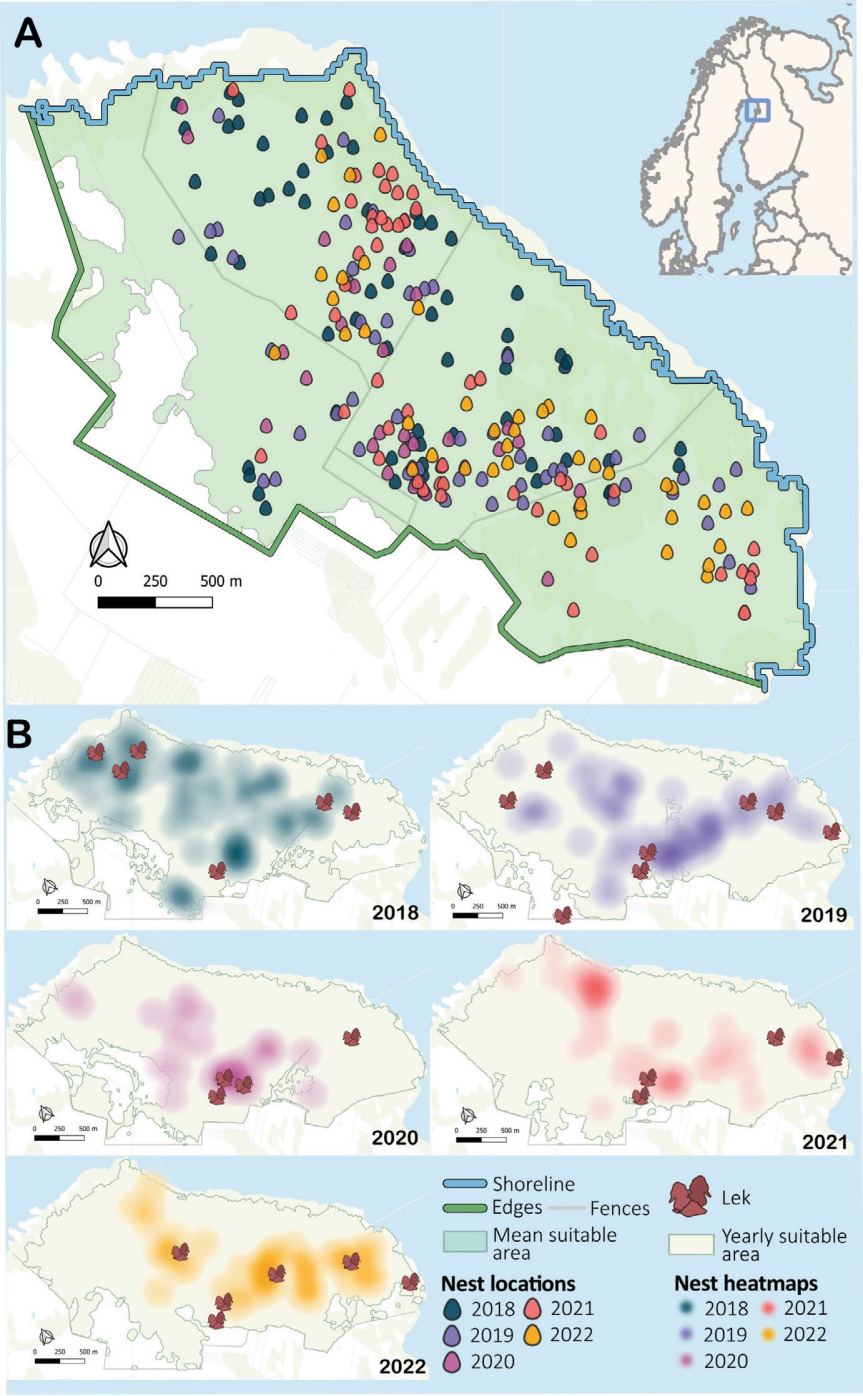
Suitable breeding areas with nest and lek distributions, shoreline, and meadow edge. (A) Field site location (blue square in map outlet) and nest locations for years 2018–2022 (colors represent years). Blue line represents the average shoreline, green line the meadow edge, and the green shaded area the suitable area for the 5 years. (B) Yearly nest distribution heat maps, active lek distributions, and suitable areas for years 2018–2022. The suitable area for 2022 was based on SAR data only.

Finally, we overlapped the two averaged measures and generated a single suitable breeding area for the entire study period, using the union of all SAR and NDVI areas (Figure 1A, Figure A1). Although the total area may be slightly larger than the actual suitable nesting area, we note that the areas with observed nest sites are quite similar to those from the satellite imagery. Hence, we used the SAR and NDVI union area to delimit the theoretical range suitable for nests. We used this area to generate randomly simulated nests for the analyses, which we then compared to the observed nest locations (see below).

### 2.4 Spatial Features of Nest Sites

We established the spatial outline of the shoreline and the meadow edge as proxy for potential flooding and predation threats and estimated the shortest distances of these spatial features to each nest. For the shoreline, we used the SAR data alone, as it clearly establishes the boundary between the meadow and shoreline. We generated a raster with the average backscatter per pixel and masked all the values that were not 0 to generate an average shoreline for the entire study time (Figure 1A). For the meadow edge, we used satellite imaging visualized in QGIS (Białowieża version 3.22, long‐term release, QGIS Association 2023). The meadow edge corresponded to the outer fence boundary of our field site (Figure 1A, green line), which marks strong vegetation changes to adjacent forests, agricultural lands, and water canals.

### 2.5 Analysis of Nest Distribution in Relation to Univariate Social and Spatial Features

#### 2.5.1 Average Nearest Neighbor Distance Analyses

We estimated whether nests were closer to or further from other nests, leks, the shoreline, and meadow edge than expected by chance, by determining the shortest distances between nests and univariate spatial features in observed versus simulated nest distributions. When comparing nest distances to other nests, we considered all nests laid in that year regardless of the nest laying and final fate dates. We used R version 4.3.3 for all statistical analyses (R Core Team 2023). We first simulated randomly distributed populations, using the *spatstat* R package (Baddeley and Turner 2005) over our designated overall suitable breeding area. For each year, we simulated 1000 nest distributions with the same number of nests as we had found at the breeding site for that year (Table 1). We estimated the average nearest neighbor distances (ANNs) of the first degree between nests by first estimating the shortest nest‐to‐nest distances for every observed and simulated nest, respectively, and then obtained an average value of these measures for each observed and simulated nest distribution, the ANNs.

For each year, we then compared the simulated ANNs to the observed ANN and calculated a pseudo *p*‐value (Equation (1), Gimond 2023) comparing the number of total simulations (*N*) to the number of simulations with a greater average ANN than the observed one (*N*_greater_). We used this one‐sided pseudo *p*‐value as the probability of false rejection of the null hypothesis of spatial randomness.

(1) $$p_{\text{pseudo}}=\frac{\min\left(N_{\text{greater}}+1,N+1-N_{\text{greater}}\right)}{N+1}$$

Finally, we estimated a combined pseudo *p*‐value based on the Stouffer's test, using the R package *metap* (Dewey 2024).

We used the same procedure to estimate whether nests were closer to or further from leks, shoreline, and meadow edges than expected by chance. When estimating the ANN between nests and leks, we took the center point of each lek and measured the distances between nests and these center points. For each year, we used the lek sites that we found active in that year. We considered shore and meadow edges as lines, and we calculated nest distances to these spatial features by measuring the shortest distance between nests and the closest point on the lines marking the shore and meadow edges. We also estimated the pseudo *p*‐values for each year.

### 2.6 Analysis of Relative Effect of Predictors Affecting Nest Distributions

We assessed the degree in which factors may influence the spatial distribution of nests, including distance to shoreline, meadow edge, other nests, and leks, with the first two as proxies for flooding and predation threat, respectively. For each year, we used the same simulated 1000 nest distributions as in the ANN analyses (see above). We used the shortest distances from each point to each spatial feature to estimate the average distances per observed and simulated nest distribution, which served to calculate the predictors: shoreline, meadow edge, nests, and leks.

To test for potential spatial autocorrelation between some of our predictors, we assessed the level of correlation between predictors using the R package *corrplot* (Wei and Simko 2021) and the Pearson correlation formula between paired samples to assess the significance of the correlation. Specifically, we assessed correlation between the predictors: (1) nest distances to shore and meadow edge; (2) nest distances to leks and shore; and (3) nest distances to leks and meadow edge. We also assessed yearly differences in correlation for the comparisons involving distance from nests to leks, as the lek locations varied between years. Assessing spatial correlation was important since, in some parts of our field site, the shoreline and meadow edge run parallel to each other; therefore, if the majority of the nests are located between these two lines, then the nest distances to edge and to shoreline could be strongly tied and nonindependent. Similarly, if a lek is located near the shore or the edge, nest distances to these spatial features might be correlated, depending on the locations of the nests. Since lek location changed throughout the years, we estimated overall correlation coefficients (Figure 3A) and also across years (Figure A7). Finally, we estimated the variance inflation factor (VIF) of the variables in the model using the R package *performance* (Lüdecke et al. 2021) to test the level of multicollinearity (i.e., level of correlation of each predictor with all other predictors).

We ran a customized generalized linear mixed model (GLMM) with a binomial distribution and logit link function and “year” as a random variable, where the response variable was whether a nest was observed or simulated. We implemented our model in a Bayesian framework using the *rstan* R package (Stan Development Team 2024). Our predictor variables were the respective average distances to the spatial features within a year. With this method, we were able to compare the extent of deviation between the observed and simulated data points using all predictors in a multivariate framework and thus test the relative effect of each predictor. The model converged with $\hat{R}$ values < 1.10 (Brooks and Gelman 1998) and we consider the effect of predictors to be clear, if the 95% credible intervals (CrI) do not overlap with 0.

## 3 Results

We located a total of 277 nests of Ruffs during the 5‐year study period of 2018–2022. Excluding nests with missing data or spatial coordinates outside of our core study area, we obtained a total of 246 nesting locations (Figure 1A, Table 1). The number and location of leks varied annually, with a minimum of four in 2020 and 2021, and a maximum of eight in 2019 (Figure 1B, Table 1). Two leks were active throughout the entire study period, whereas two other leks were active in 3 of 5 years. We estimated the overall egg‐laying period for the 5 years based on the earliest and latest start dates to be between the 14th of May and the 25th of June (Table 1).

### 3.1 Spatial Correlation

**FIGURE 2:**
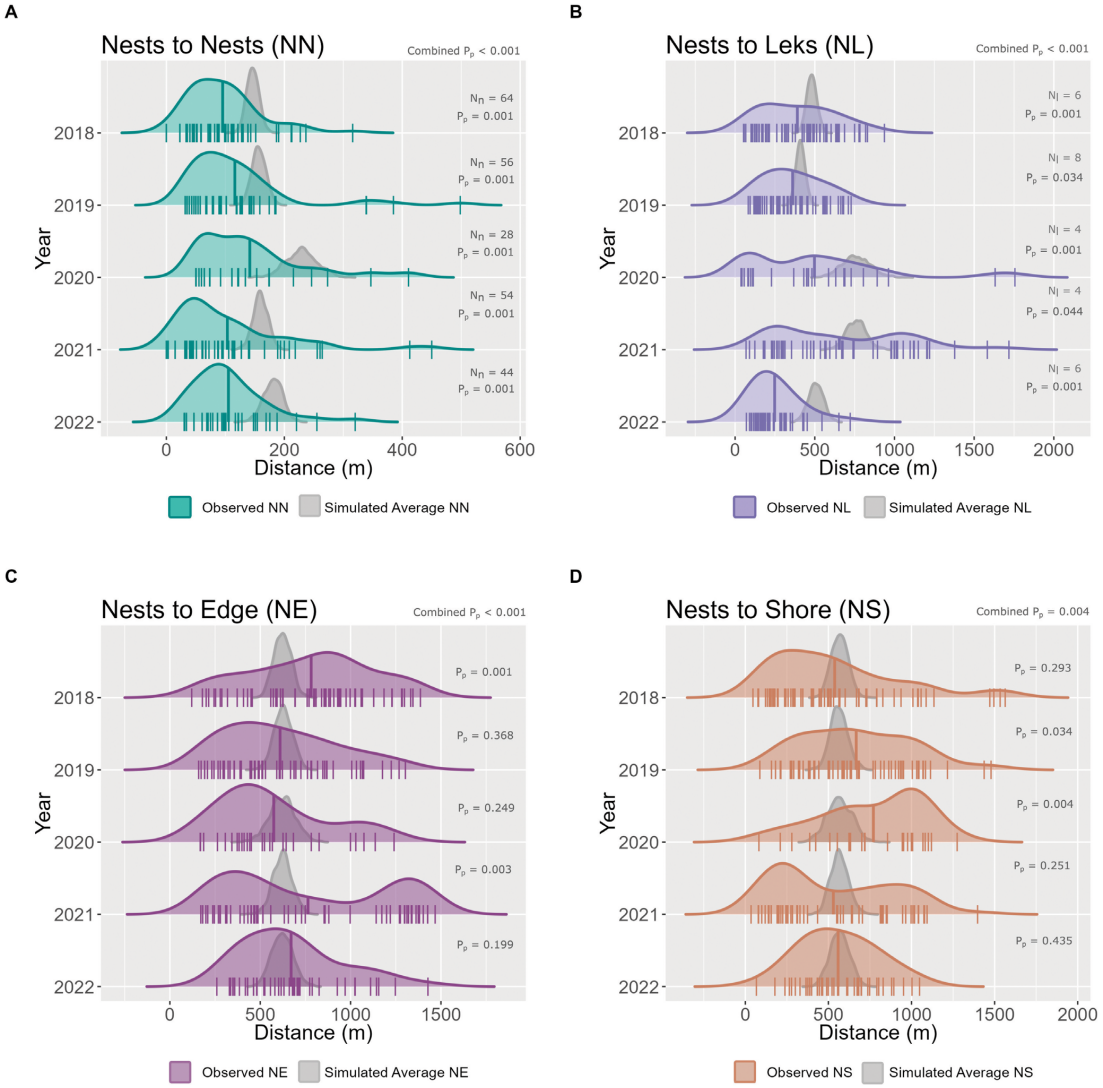
Average nearest neighbor (ANN) distances between nests and four other spatial features: (A) nests of other reeves, (B) leks, (C) meadow edge, and (D) shore. All plots show observed (colored) and simulated (gray) nearest neighbor distance distributions to one of the spatial features for the years 2018–2022. The short vertical lines of the colored annual distributions show the observed distances, long vertical lines represent observed annual ANN distances; *N*_*n*_ in A indicates the number of nests, and *N*_*l*_ in B indicates the number of leks per year. For each scenario, singular pseudo *p*‐values (P*p*) and a combined pseudo *p*‐value are provided. Simulated ANN distance distributions show the range of ANN distances for 1000 distributions each using the same number of nests that we found in each year.

Ruff nests were located between 3 and 498 m away from other nests (x̄ = 109 m, Figure 2A), and 38 and 1756 m away from leks (x̄ = 428 m, Figure 2B). Nests were between 34 and 1563 m from the shoreline (x̄ = 595 m, Figure 2D) and between 120 and 1467 m from the meadow edge (x̄ = 696 m, Figure 2C).

**FIGURE 3:**
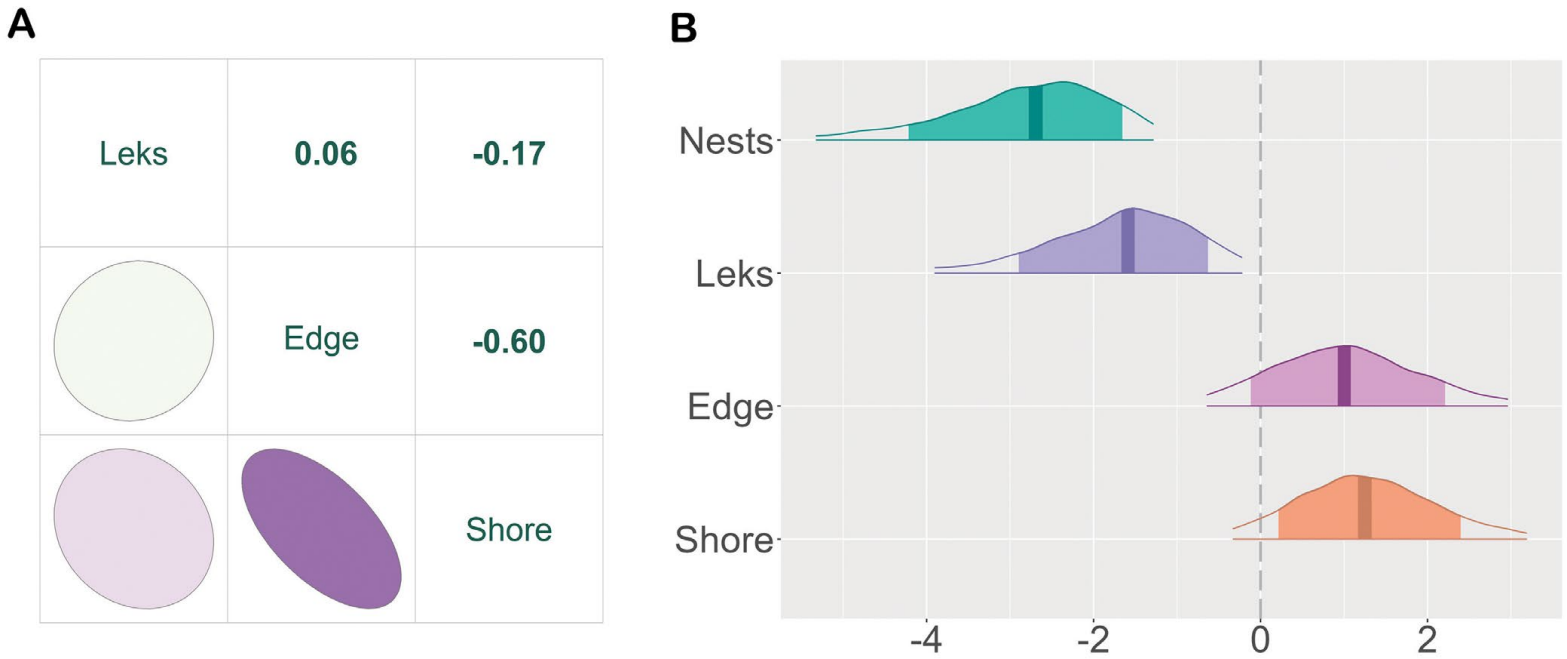
Model results for factors affecting nest locations. (A) Correlation matrix between leks, meadow edge, and shore to nest distances; all Pearson's correlations are significantly different from zero. (B) Parameter effect plots for nests, leks, meadow edge, and shore to nest distances, with the outer lines indicating the 95% credible intervals, and the 80% credible intervals indicated by colored areas.

The distances between nests to meadow edge and nests to the shoreline were negatively correlated with each other (Pearson's correlation coefficient: −0.60, *p* < 0.01, Figure 3A; with a range of −0.58 to −0.63 across years, Figure A7), indicating that a substantial number of nests were located at the part of the field site where the shoreline and the meadow edge run parallel. Distance from nests to leks showed a weak correlation with the other two spatial predictors (Pearson's correlation coefficient for shoreline: −0.17, *p* < 0.01, with a range of −0.42 to 0.13 across years; Pearson's correlation coefficient for meadow edge: 0.06, *p* < 0.01, with a range of −0.3 to 0.25 across years; Figure 3A, Figure A7), which indicates that nest to lek distances were mostly independent from nest to meadow edge or shoreline distances. There was no evidence for multicollinearity between predictors in the GLMM; all VIFs were < 2.

### 3.2 Nest Distances to Individual Spatial Features

The observed average nest‐to‐nest distances were consistently smaller than the distribution of simulated values, indicating that nests are located closer to each other than expected by chance (${\mathrm{ANN}}_{2018}^{\mathrm{NN}}$ = 95.4 m, ${\mathrm{ANN}}_{2019}^{\mathrm{NN}}$ = 116.1 m, ${\mathrm{ANN}}_{2020}^{\mathrm{NN}}$ = 141.5 m, ${\mathrm{ANN}}_{2021}^{\mathrm{NN}}$ = 103.4 m, ${\mathrm{ANN}}_{2022}^{\mathrm{NN}}$ = 105.3 m, all *p*_pseudo_ < 0.05, Figure 2A). The difference between the observed and the simulated ANNs ranged from 1.5 to 169 m, with an average of 62.3 m.

Similarly, the ANNs in the nest‐to‐lek comparison varied from year to year (${\mathrm{ANN}}_{2018}^{\mathrm{NL}}$ = 390.6 m, ${\mathrm{ANN}}_{2019}^{\mathrm{NL}}$ = 361.1 m, ${\mathrm{ANN}}_{2020}^{\mathrm{NL}}$ = 497.9 m, ${\mathrm{ANN}}_{2021}^{\mathrm{NL}}$ = 653.6 m, ${\mathrm{ANN}}_{2022}^{\mathrm{NL}}$ = 248.2 m, Figure 2B). The nests tended to be closer to leks than expected by chance (all *p*_pseudo_ < 0.05). The difference between the observed and the simulated ANNs ranged from −82 m (in which case the observed ANN was larger than the simulated ANN) to 589 m, with an average of 154 m.

In years with fewer nests (e.g., 2020) and fewer leks (e.g., 2020, 2021), the distributions of the simulated ANNs are wider for both comparisons (Figure 2B). Similarly, the observed nearest neighbor distances between nests and leks in 2020 and 2021 showed a much broader distribution (Figure 2B).

Whether observed nests were closer to or further away from the shoreline and the meadow edge than simulated ones varied among years. Observed nests were further away from the meadow edge than simulated ones in 2018 and 2021 (${\mathrm{ANN}}_{2018}^{\mathrm{NE}}$ = 781.9 m, ${\mathrm{ANN}}_{2021}^{\mathrm{NE}}$ = 764.3 m, all *p*_pseudo_ < 0.05, Figure 2C); however, the observed ANN overlapped considerably with the simulated ANN distributions in 2019, 2020, and 2022 (${\mathrm{ANN}}_{2019}^{\mathrm{NE}}$ = 609.9 m, ${\mathrm{ANN}}_{2020}^{\mathrm{NE}}$ = 574.8 m, ${\mathrm{ANN}}_{2022}^{\mathrm{NE}}$ = 670.8 m, all *p*_pseudo_ > 0.1, Figure 2C). The difference between the observed and the simulated ANNs ranged from −336 to +261 m, with an average of −56.9 m. As for the distance to the shoreline, observed nests were further away from the shoreline than simulated ones in 2019 and 2020 (${\mathrm{ANN}}_{2019}^{\mathrm{NS}}$ = 667.1 m, ${\mathrm{ANN}}_{2020}^{\mathrm{NS}}$ = 770.7 m, *p*_pseudo_ < 0.05, Figure 2D), whereas the observed ANN overlapped considerably with the distribution of the simulated ANNs in the rest of the years (${\mathrm{ANN}}_{2018}^{\mathrm{NS}}$ = 537.7 m, ${\mathrm{ANN}}_{2021}^{\mathrm{NS}}$ = 529.7 m, ${\mathrm{ANN}}_{2022}^{\mathrm{NS}}$ = 558.4 m, *p*_pseudo_ > 0.2, Figure 2D). The difference between the observed and the simulated ANNs between nests and the meadow edge ranged from −414 to 232 m, with an average of −46.1 m.

Across all years, combined pseudo *p*‐values for each comparison were < 0.05 (Figure 2) suggesting that each of the four spatial features that we tested affected nest distributions.

### 3.3 Relative Influence of Factors Affecting Nesting Distributions

The results of our binomial GLMM indicated the clearest difference between the locations of the simulated and observed data points in the predictor distances to other nests, as Ruff females placed nests closer to other nests than expected by chance (x̄ = −2.8 with 95% CrI: −5.3 to −1.3; Table 3; Figure 3B). The second strongest effect was the distance to leks, which were also shorter than expected by chance (x̄ = −1.7 with 95% CrI: −3.9 to −0.2; Table 3; Figure 3B).

**TABLE 3:** Results of a generalized linear mixed model (GLMM) for five factors affecting nest locations.

Parameter	Estimate mean	Estimate SD	95% CrI	ESS
(Intercept)	−15.6	3.7	[−24.5, −10.3]	1355
Nests	−2.8	1	[−5.3, −1.3]	1739
Leks	−1.7	0.9	[−3.9, −0.2]	2423
Edge	1	0.9	[−0.6, 3]	2345
Shore	1.3	0.9	[−0.3, 3.2]	2618
σ_year_	0.9	2	[0, 5.8]	3155

*Note:* The parameter estimates mean, standard deviation (SD), 95% credible intervals (95% CrI) and effective sample sizes (ESS) are for the binomial model considering the effects of the scaled nest distance to other nests, leks, meadow edge and shoreline, and year as a random factor (with σ_year_ as the standard deviation explained by the random factor year). All variables had an $\hat{R}$ of 1.

**FIGURE A6:**
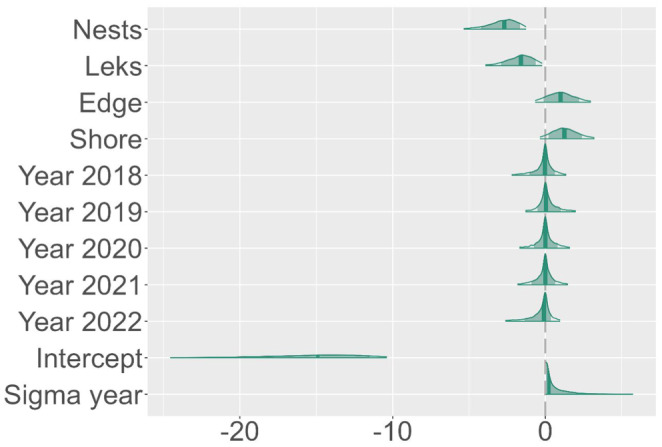
Posterior distributions of factors affecting nest locations from a Bayesian model. Parameter effect plots for nests, leks, edge, and shore to nests distances, year effects, and intercept, with the outer lines indicating the 95% credible intervals and 80% credible intervals indicated by colored areas.

Reeves tended to place nests further away from the meadow edge than expected from the randomly simulated nests (x̄ = 1 with 95% CrI: −0.6 to 3, Table 3; Figure 3B). They also tended to place nests further from the shoreline than the simulated nests (x̄ = 1.3 with 95% CrI: −0.3 to 3.2, Table 3; Figure 3B). However, the 95% CrI of distance to the shore and meadow edge overlapped with zero, indicating that the effects were not statistically clear. The random variance of year was small (σ_year_: 0.9 with 95% CrI: 0–5.8, Table 3, Figure A6).

## 4 Discussion

We examined the nest distribution of Ruffs in relation to social and environmental features of the landscape. We found that nest distributions were not random with regard to social and environmental cues, with females nesting closer to leks and nests of other females than expected by chance. Despite substantial annual variation, nests tended to be further away from both meadow edges and shorelines, two habitat features that are typically associated with threats such as predation and flooding. The ANN analyses with univariate social cues as predictors of annual nest distributions showed that Ruff nests have some level of spatial aggregation among themselves and around leks. In all study years, the average distance among nests and between leks and nests was consistently shorter than between simulated nests. The spatial relationships were confirmed by the GLMM, in which distance to other nests and leks showed the clearest difference between the observed and simulated data points. Our results suggest that reeves either consider social features of the habitat such as the proximity to leks and nests of neighboring females when selecting their own nest location, or that their selection of the nest location is based on other underlying habitat parameters that then lead to aggregation patterns.

Nest aggregations may be driven by resource availability or use of social information, such as by birds copying the breeding‐site choices of conspecifics (Doligez et al. 2003; Szymkowiak et al. 2016), resulting in clustered nest distributions. An alternative explanation is that nest aggregations are shaped by other environmental features that we did not measure. In other waders, such as the American Golden Plover ( *Pluvialis dominica* ), Dunlin ( *Calidris alpina* ), Pectoral Sandpiper ( *C. melanotos* ) or the Semipalmated Sandpiper ( *C. pusilla* ), nest distributions were strongly associated with environmental factors, such as tundra wetness or level of microrelief. However, nests in these species tended to be located further away from conspecific nests than expected by chance (Cunningham et al. 2016; Freeman et al. 2023).

Nest aggregations can also be beneficial to reduce predation risk, especially when the community includes species with active nest defense or heightened vigilance (Hamilton 1971; Götmark and Andersson 1984; Perry and Andersen 2003). Nesting close to waders with strong anti‐predatory responses has beneficial effects on nest survival of other ground‐breeding birds and can even induce elevated vigilance responses in small mammals (Waterman and Mai 2020; Gupta et al. 2022). Reeves rely largely on the camouflage of their nests and only rarely alarm during incubation (Scheufler and Stiefel 1985); however, they often nest near sentinel birds such as Black‐tailed Godwits ( *Limosa limosa* ), Curlews ( *Numenius arquata* ), and Northern Lapwings ( *Vanellus vanellus* ) (Gochfeld 1984, Belfín et al. 2024, personal observations). Furthermore, especially in the early stages of incubation, females tend to flush from their nest when they detect a predator at a long distance (Scheufler and Stiefel 1985, personal observations). Nearby incubating females may use this flight behavior as a cue to leave the nest themselves, recognizing the presence of a potential predator in the area.

The second major social predictor of nests was proximity to leks. We found that observed nest‐to‐lek distances were consistently shorter than the corresponding distances using simulated nests in suitable nesting habitats. The spatial relationship between nest and lek locations is species dependent but can be relevant for conservation management and understanding the evolution of lekking. Our findings suggest that the presence of leks can be used as a proxy to identify important nesting habitats and hence select areas for conservation management. In other lekking birds such as the Great Bustard ( *Otis tarda* ), leks were also used to identify nesting areas, although the presence of leks may have biased the nest search, as subsequently Great Bustard females were also found nesting in other areas (Magaña et al. 2011).

The relationship between nest and lek locations is not well understood, particularly which factor takes precedence (Beehler and Foster 1988). Information on current lek and past nest locations may be important social cues for each sex to identify adequate settling areas. Our correlative analyses cannot determine the initial cause of the settlement and its consequence on future settlement; but our results suggest that both nest and lek placement are potentially important social cues during habitat selection in Ruffs. Lekking males may preferably gather in areas of high female presence (Bradbury and Gibson 1983; Beehler and Foster 1988; Höglund et al. 1998) or place leks in areas where females choose to breed. Ruff males arrive at the breeding grounds earlier and establish leks prior to female arrival (Höglund and Alatalo 1995), and therefore cannot rely on the females' locations of the current year to find a place to settle the lek. Forming leks in areas that have provided favorable nesting conditions for females in previous years should be beneficial for males and provide newly breeding females with cues about habitat quality (*hotspot* hypothesis, Bradbury and Gibson 1983, Beehler and Foster 1988). Returning males may hence use densities of breeding reeves that they experienced in previous years to choose profitable lekking sites in the consecutive breeding season or try to identify favorable nesting habitat, which would lead to a higher female encounter rate (Höglund et al. 1998). Thus, females may be able to use lek locations as cues to identify suitable breeding areas, which would result in a positive feedback dynamic that reinforces the proximity of nest and lek locations after the initial establishment of breeding activity in a local area. Leks serving as favorable nest site cues for females have been explored in the Great Snipe (*Gallinago media*), another lekking wader, where males place their leks in areas with high food abundance and females nest close to leks, assuring them close access to food resources (Løfaldli et al. 1992; Korniluk et al. 2021).

Additionally, females could also benefit from nesting close to lekking areas by means of leks acting as sentinels or decoys. The *sentinel/decoy model* for lek evolution postulates that females nesting close to lekking areas benefit from lower predation risk as lekking males will signal the approach of predators (“sentinel”), or predators will be attracted to leks (“decoy”) but not search intensively the surrounding areas (Phillips 1990).

Not all females rely on the presence of active leks to choose their nest sites. Remarkably, in 2020 and 2021, we found nests in the northwestern parts of our field site despite an absence of leks in this area. In both years, the observed distance distributions were particularly wide with some nests being very far from active leks. It is unlikely that we missed sustained lekking activity in these areas since we surveyed the area regularly throughout the breeding season. Returning females to successful nest locations from previous years could potentially explain why we still found nests at the location in the following years, despite the absence of active leks. Interestingly, the nest distributions of the subsequent years seemed to slowly shift toward the eastern parts of our field site. We have observed a gradual deterioration of the vegetation in the western parts of the study site where the early lek sites were abandoned over time, which could explain the gradual move of both sexes from the area. Shifting distributions suggest that indeed vegetation and availability of suitable habitat are key determinants of both lek and nest location (Höglund et al. 1998) highlighting the importance of appropriate meadow management.

**FIGURE A7:**
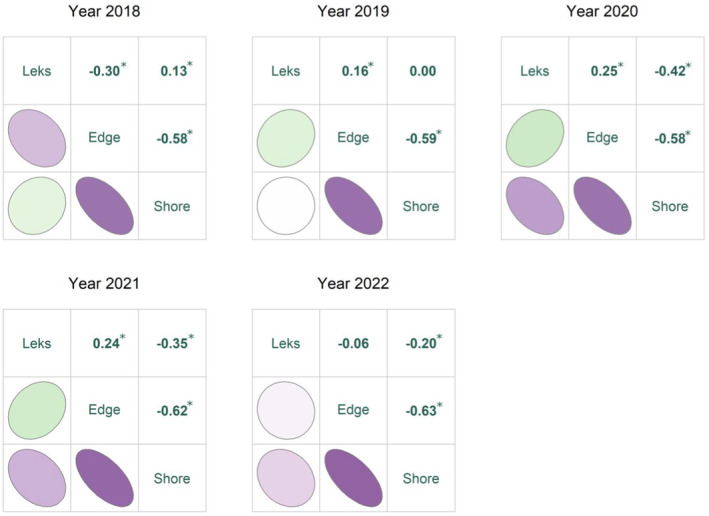
Model results for factors affecting nest locations. A. Correlation matrix between distance to leks, edge, and shore. The significant Pearson's correlations are indicated with an asterisk (*).

The effect of distance to the meadow edge and shoreline on nest locations was less clear and showed considerable yearly variation. Edge effects can increase predator presence and the risk of nest failure (Kaasiku et al. 2022). In some years, Ruff nests were further from meadow edges than expected by chance which suggests that females may avoid edge areas when choosing a nest site because of increased predation risk. Furthermore, in some years, nests were located further away from the shoreline than expected by chance. Nesting close to the shoreline can be beneficial for the survival of young chicks because they may hatch close to feeding sites, but on the other hand, the proximity to the shoreline represents a common risk for wader nests (Van de Pol et al. 2010; Koivula et al. 2025) as changes in tides and water level ground their destruction and abandonment. When flooding occurs regularly, some birds may develop adaptations such as breeding at times where tides are lowest (Plaschke et al. 2019). However, due to ongoing climate change, coastal wetlands are now exposed to wind floods and sea level rise. Both the increased amplitude and frequency of flooding events result in higher losses of nests and chicks (Erwin et al. 2006; Van de Pol et al. 2010; Bailey et al. 2017; Koivula et al. 2025). Therefore, nesting close to the shore may have implications for offspring survival and female reproductive fitness; hence, females could use distance to the shoreline as a proxy for nest success. The effects of closeness to the shore and meadow edges were not consistent throughout the years and may have been further blurred by the detected inverse correlation between the distances of nest locations to the shoreline and to the meadow edge, respectively (Figure 3A, Figure A7). In many parts of the field site, the two edges run parallel to each other; therefore, it is hard to be far from the shore and at the same time far from the meadow edge, which would explain why the relative importance of the two seemed to change yearly. The GLMM did not show an effect of year (Figure A6), suggesting that at least the social cues, distance to other nests and distance to leks, were important for nest location during all study years (Figure 2A,B).

### 4.1 Implications for Conservation

Coastal meadows host numerous species of breeding waders, many of conservation concern. Using remote sensing and considering species requirements for different sexes or life stages can help to decipher species optima on larger scales. Ruffs provide an interesting model for studies in ecology, evolutionary, and behavioral biology due to their high intraspecific variation, diverse sex roles, and negative population trends (Hugie and Lank 1997; Lank et al. 2013; Giraldo‐Deck et al. 2022). Previous Ruff research has mostly focused on flamboyant males while female behaviors remained largely unexplored (but see Giraldo‐Deck et al. 2022). Studying the “female‐perspective” and how females choose their nest sites improves our understanding of the dynamics of Ruff populations and can help to create conditions for population growth. The apparent importance of lek locations for nesting sites and the ease of identifying leks thanks to the conspicuous male displays implicate that lekking areas can be used as a proxy to recognize potential nesting areas with high conservation and management priority. If lek locations are influencing nest distributions, creating attractive lekking sites in good nesting habitat could improve the breeding success and, in turn, population growth of this species. Future research should investigate the effects of aggregations and social and environmental spatial habitat features on nest survival and strive for a better understanding of optimal habitat requirements of both males and females.

## Author Contributions

**Hanna Algora:** conceptualization (lead), data curation (lead), formal analysis (lead), investigation (equal), methodology (lead), software (lead), visualization (lead), writing – original draft (lead). **James D. M. Tolliver:** investigation (equal), writing – review and editing (supporting). **Veli‐Matti Pakanen:** funding acquisition (equal), investigation (equal), writing – review and editing (supporting). **Krisztina Kupán:** formal analysis (supporting), writing – review and editing (supporting). **Jelena Belojević:** investigation (supporting), writing – review and editing (supporting). **Nelli Rönkä:** funding acquisition (equal), investigation (equal), writing – review and editing (supporting). **Clemens Küpper:** conceptualization (equal), funding acquisition (lead), investigation (equal), supervision (lead), writing – review and editing (lead). **Kari Koivula:** conceptualization (equal), funding acquisition (lead), investigation (equal), supervision (lead), writing – review and editing (lead).

## Conflicts of Interest

The authors declare no conflicts of interest.

## Data Availability

All data generated or analyzed during this study are openly available in Zenodo at https://doi.org/10.5281/zenodo.12935989. The dataset and scripts necessary for reproducing the analyses presented in this study are also included. Researchers and interested parties are encouraged to access and use these data in accordance with the terms of the Creative Commons Attribution 4.0 International license.

## Appendix A

**TABLE A1:** NDVI image metadata.

Name	Mission	Level	Sensing date‐time	Orbit	Tile	% Cloud
S2B_MSIL2A_20180514T101029_N0207_R022_T34WFT_20180514T122533.SAFE	2B	2A	2018‐05‐14 10:10:29 UTC	022	34WFT	6.63
S2A_MSIL2A_20180516T100031_N0207_R122_T34WFT_20180516T111147.SAFE	2A	2A	2018‐05‐16 10:00:31 UTC	122	34WFT	4.52
S2B_MSIL2A_20180517T102019_N0207_R065_T34WFT_20180517T122617.SAFE	2B	2A	2018‐05‐17 10:20:19 UTC	065	34WFT	100
S2B_MSIL2A_20180518T095029_N0207_R079_T34WFT_20180518T115734.SAFE	2B	2A	2018‐05‐18 09:50:29 UTC	079	34WFT	2.88
S2A_MSIL2A_20180519T101031_N0207_R022_T34WFT_20180519T122503.SAFE	2A	2A	2018‐05‐19 10:10:31 UTC	022	34WFT	1.77
S2B_MSIL2A_20180521T100029_N0207_R122_T34WFT_20180521T120149.SAFE	2B	2A	2018‐05‐21 10:00:29 UTC	122	34WFT	1.56
S2A_MSIL2A_20180522T102031_N0207_R065_T34WFT_20180522T123546.SAFE	2A	2A	2018‐05‐22 10:20:31 UTC	065	34WFT	100
S2A_MSIL2A_20180523T095031_N0208_R079_T34WFT_20180523T144510.SAFE	2A	2A	2018‐05‐23 09:50:31 UTC	079	34WFT	1.2
S2B_MSIL2A_20180524T101019_N0208_R022_T34WFT_20180524T145442.SAFE	2B	2A	2018‐05‐24 10:10:19 UTC	022	34WFT	100
S2A_MSIL2A_20180526T100031_N0208_R122_T34WFT_20180526T140955.SAFE	2A	2A	2018‐05‐26 10:00:31 UTC	122	34WFT	0.41
S2A_MSIL2A_20180529T101031_N0208_R022_T34WFT_20180529T104936.SAFE	2A	2A	2018‐05‐29 10:10:31 UTC	022	34WFT	45.75
S2B_MSIL2A_20180531T100029_N0208_R122_T34WFT_20180531T123243.SAFE	2B	2A	2018‐05‐31 10:00:29 UTC	122	34WFT	0.57
S2A_MSIL2A_20180601T102021_N0208_R065_T34WFT_20180601T132906.SAFE	2A	2A	2018‐06‐01 10:20:21 UTC	065	34WFT	100
S2A_MSIL2A_20180602T095031_N0208_R079_T34WFT_20180602T122748.SAFE	2A	2A	2018‐06‐02 09:50:31 UTC	079	34WFT	0.34
S2B_MSIL2A_20180603T101019_N0208_R022_T34WFT_20180603T115150.SAFE	2B	2A	2018‐06‐03 10:10:19 UTC	022	34WFT	100
S2A_MSIL2A_20180605T100031_N0208_R122_T34WFT_20180605T111732.SAFE	2A	2A	2018‐06‐05 10:00:31 UTC	122	34WFT	40.31
S2B_MSIL2A_20180606T102019_N0208_R065_T34WFT_20180606T161417.SAFE	2B	2A	2018‐06‐06 10:20:19 UTC	065	34WFT	100
S2B_MSIL2A_20180607T095029_N0208_R079_T34WFT_20180607T122324.SAFE	2B	2A	2018‐06‐07 09:50:29 UTC	079	34WFT	100
S2A_MSIL2A_20180608T101021_N0208_R022_T34WFT_20180608T131739.SAFE	2A	2A	2018‐06‐08 10:10:21 UTC	022	34WFT	2.55
S2B_MSIL2A_20180610T100029_N0208_R122_T34WFT_20180610T124206.SAFE	2B	2A	2018‐06‐10 10:00:29 UTC	122	34WFT	100
S2A_MSIL2A_20180611T102021_N0208_R065_T34WFT_20180611T125440.SAFE	2A	2A	2018‐06‐11 10:20:21 UTC	065	34WFT	100
S2A_MSIL2A_20180612T095031_N0208_R079_T34WFT_20180612T123228.SAFE	2A	2A	2018‐06‐12 09:50:31 UTC	079	34WFT	0.59
S2B_MSIL2A_20180613T101019_N0208_R022_T34WFT_20180613T115316.SAFE	2B	2A	2018‐06‐13 10:10:19 UTC	022	34WFT	29.13
S2A_MSIL2A_20180615T100031_N0208_R122_T34WFT_20180615T113219.SAFE	2A	2A	2018‐06‐15 10:00:31 UTC	122	34WFT	100
S2B_MSIL2A_20180616T102019_N0208_R065_T34WFT_20180616T162410.SAFE	2B	2A	2018‐06‐16 10:20:19 UTC	065	34WFT	100
S2B_MSIL2A_20180617T095029_N0208_R079_T34WFT_20180617T124450.SAFE	2B	2A	2018‐06‐17 09:50:29 UTC	079	34WFT	0.81
S2A_MSIL2A_20180618T101021_N0208_R022_T34WFT_20180618T131431.SAFE	2A	2A	2018‐06‐18 10:10:21 UTC	022	34WFT	53.24
S2B_MSIL2A_20180620T100029_N0208_R122_T34WFT_20180620T154356.SAFE	2B	2A	2018‐06‐20 10:00:29 UTC	122	34WFT	0.46
S2A_MSIL2A_20180621T102021_N0208_R065_T34WFT_20180621T131841.SAFE	2A	2A	2018‐06‐21 10:20:21 UTC	065	34WFT	100
S2A_MSIL2A_20180622T095031_N0208_R079_T34WFT_20180622T110702.SAFE	2A	2A	2018‐06‐22 09:50:31 UTC	079	34WFT	100
S2B_MSIL2A_20180623T101029_N0208_R022_T34WFT_20180623T131922.SAFE	2B	2A	2018‐06‐23 10:10:29 UTC	022	34WFT	100
S2A_MSIL2A_20180625T100031_N0208_R122_T34WFT_20180625T143253.SAFE	2A	2A	2018‐06‐25 10:00:31 UTC	122	34WFT	1.18
S2A_MSIL2A_20190514T101031_N0212_R022_T34WFT_20190514T125603.SAFE	2A	2A	2019‐05‐14 10:10:31 UTC	022	34WFT	35.7
S2B_MSIL2A_20190516T100039_N0212_R122_T34WFT_20190516T131308.SAFE	2B	2A	2019‐05‐16 10:00:39 UTC	122	34WFT	87.31
S2A_MSIL2A_20190517T102031_N0212_R065_T34WFT_20190517T113806.SAFE	2A	2A	2019‐05‐17 10:20:31 UTC	065	34WFT	100
S2A_MSIL2A_20190518T095031_N0212_R079_T34WFT_20190518T124045.SAFE	2A	2A	2019‐05‐18 09:50:31 UTC	079	34WFT	9.66
S2B_MSIL2A_20190519T101039_N0212_R022_T34WFT_20190519T132053.SAFE	2B	2A	2019‐05‐19 10:10:39 UTC	022	34WFT	5.11
S2A_MSIL2A_20190521T100031_N0212_R122_T34WFT_20190521T130611.SAFE	2A	2A	2019‐05‐21 10:00:31 UTC	122	34WFT	56.93
S2B_MSIL2A_20190523T095039_N0212_R079_T34WFT_20190523T113546.SAFE	2B	2A	2019‐05‐23 09:50:39 UTC	079	34WFT	100
S2A_MSIL2A_20190524T101031_N0212_R022_T34WFT_20190524T114010.SAFE	2A	2A	2019‐05‐24 10:10:31 UTC	022	34WFT	100
S2B_MSIL2A_20190526T100039_N0212_R122_T34WFT_20190526T130259.SAFE	2B	2A	2019‐05‐26 10:00:39 UTC	122	34WFT	100
S2A_MSIL2A_20190527T102031_N0212_R065_T34WFT_20190527T145156.SAFE	2A	2A	2019‐05‐27 10:20:31 UTC	065	34WFT	100
S2A_MSIL2A_20190528T095031_N0212_R079_T34WFT_20190528T120427.SAFE	2A	2A	2019‐05‐28 09:50:31 UTC	079	34WFT	97.03
S2B_MSIL2A_20190529T101039_N0212_R022_T34WFT_20190529T130331.SAFE	2B	2A	2019‐05‐29 10:10:39 UTC	022	34WFT	100
S2A_MSIL2A_20190531T100031_N0212_R122_T34WFT_20190531T113301.SAFE	2A	2A	2019‐05‐31 10:00:31 UTC	122	34WFT	99.83
S2B_MSIL2A_20190601T102029_N0212_R065_T34WFT_20190601T135040.SAFE	2B	2A	2019‐06‐01 10:20:29 UTC	065	34WFT	100
S2B_MSIL2A_20190602T095039_N0212_R079_T34WFT_20190602T122701.SAFE	2B	2A	2019‐06‐02 09:50:39 UTC	079	34WFT	1.64
S2A_MSIL2A_20190603T101031_N0212_R022_T34WFT_20190603T114652.SAFE	2A	2A	2019‐06‐03 10:10:31 UTC	022	34WFT	100
S2B_MSIL2A_20190605T100039_N0212_R122_T34WFT_20190605T131920.SAFE	2B	2A	2019‐06‐05 10:00:39 UTC	122	34WFT	1.53
S2A_MSIL2A_20190606T102031_N0212_R065_T34WFT_20190606T124321.SAFE	2A	2A	2019‐06‐06 10:20:31 UTC	065	34WFT	100
S2A_MSIL2A_20190607T095031_N0212_R079_T34WFT_20190607T112230.SAFE	2A	2A	2019‐06‐07 09:50:31 UTC	079	34WFT	92.47
S2B_MSIL2A_20190608T101029_N0212_R022_T34WFT_20190608T132159.SAFE	2B	2A	2019‐06‐08 10:10:29 UTC	022	34WFT	45.06
S2A_MSIL2A_20190610T100031_N0212_R122_T34WFT_20190610T112728.SAFE	2A	2A	2019‐06‐10 10:00:31 UTC	122	34WFT	1.77
S2B_MSIL2A_20190611T102029_N0212_R065_T34WFT_20190611T134732.SAFE	2B	2A	2019‐06‐11 10:20:29 UTC	065	34WFT	100
S2B_MSIL2A_20190612T095039_N0212_R079_T34WFT_20190612T124245.SAFE	2B	2A	2019‐06‐12 09:50:39 UTC	079	34WFT	16.14
S2A_MSIL2A_20190613T101031_N0212_R022_T34WFT_20190614T125329.SAFE	2A	2A	2019‐06‐13 10:10:31 UTC	022	34WFT	92.93
S2B_MSIL2A_20190615T100039_N0212_R122_T34WFT_20190615T130417.SAFE	2B	2A	2019‐06‐15 10:00:39 UTC	122	34WFT	0.91
S2A_MSIL2A_20190616T102031_N0212_R065_T34WFT_20190616T133105.SAFE	2A	2A	2019‐06‐16 10:20:31 UTC	065	34WFT	100
S2A_MSIL2A_20190617T095031_N0212_R079_T34WFT_20190617T112801.SAFE	2A	2A	2019‐06‐17 09:50:31 UTC	079	34WFT	71.72
S2B_MSIL2A_20190618T101029_N0212_R022_T34WFT_20190618T131049.SAFE	2B	2A	2019‐06‐18 10:10:29 UTC	022	34WFT	0.37
S2A_MSIL2A_20190620T100031_N0212_R122_T34WFT_20190620T131720.SAFE	2A	2A	2019‐06‐20 10:00:31 UTC	122	34WFT	100
S2B_MSIL2A_20190621T102029_N0212_R065_T34WFT_20190621T133555.SAFE	2B	2A	2019‐06‐21 10:20:29 UTC	065	34WFT	100
S2B_MSIL2A_20190622T095039_N0212_R079_T34WFT_20190622T123513.SAFE	2B	2A	2019‐06‐22 09:50:39 UTC	079	34WFT	100
S2A_MSIL2A_20190623T101031_N0212_R022_T34WFT_20190623T132509.SAFE	2A	2A	2019‐06‐23 10:10:31 UTC	022	34WFT	100
S2B_MSIL2A_20190625T100039_N0212_R122_T34WFT_20190625T131010.SAFE	2B	2A	2019‐06‐25 10:00:39 UTC	122	34WFT	0.4
S2A_MSIL2A_20200515T100031_N0214_R122_T34WFT_20200515T141839.SAFE	2A	2A	2020‐05‐15 10:00:31 UTC	122	34WFT	70.19
S2B_MSIL2A_20200516T101559_N0214_R065_T34WFT_20200516T134122.SAFE	2B	2A	2020‐05‐16 10:15:59 UTC	065	34WFT	100
S2B_MSIL2A_20200517T095029_N0214_R079_T34WFT_20200517T123047.SAFE	2B	2A	2020‐05‐17 09:50:29 UTC	079	34WFT	100
S2A_MSIL2A_20200518T101031_N0214_R022_T34WFT_20200518T114205.SAFE	2A	2A	2020‐05‐18 10:10:31 UTC	022	34WFT	6.75
S2B_MSIL2A_20200520T100029_N0214_R122_T34WFT_20200520T125639.SAFE	2B	2A	2020‐05‐20 10:00:29 UTC	122	34WFT	95.05
S2A_MSIL2A_20200521T102031_N0214_R065_T34WFT_20200521T110235.SAFE	2A	2A	2020‐05‐21 10:20:31 UTC	065	34WFT	100
S2A_MSIL2A_20200522T095041_N0214_R079_T34WFT_20200522T123919.SAFE	2A	2A	2020‐05‐22 09:50:41 UTC	079	34WFT	3.09
S2B_MSIL2A_20200523T100559_N0214_R022_T34WFT_20200523T132956.SAFE	2B	2A	2020‐05‐23 10:05:59 UTC	022	34WFT	2.31
S2A_MSIL2A_20200525T100031_N0214_R122_T34WFT_20200525T114447.SAFE	2A	2A	2020‐05‐25 10:00:31 UTC	122	34WFT	2.51
S2B_MSIL2A_20200526T101559_N0214_R065_T34WFT_20200526T135521.SAFE	2B	2A	2020‐05‐26 10:15:59 UTC	065	34WFT	100
S2B_MSIL2A_20200527T095029_N0214_R079_T34WFT_20200527T124319.SAFE	2B	2A	2020‐05‐27 09:50:29 UTC	079	34WFT	100
S2A_MSIL2A_20200528T101031_N0214_R022_T34WFT_20200528T105201.SAFE	2A	2A	2020‐05‐28 10:10:31 UTC	022	34WFT	2.92
S2B_MSIL2A_20200530T100029_N0214_R122_T34WFT_20200530T123822.SAFE	2B	2A	2020‐05‐30 10:00:29 UTC	122	34WFT	2.72
S2A_MSIL2A_20200531T102031_N0214_R065_T34WFT_20200531T125547.SAFE	2A	2A	2020‐05‐31 10:20:31 UTC	065	34WFT	100
S2A_MSIL2A_20200601T095041_N0214_R079_T34WFT_20200601T112207.SAFE	2A	2A	2020‐06‐01 09:50:41 UTC	079	34WFT	4.08
S2B_MSIL2A_20200602T100559_N0214_R022_T34WFT_20200602T135324.SAFE	2B	2A	2020‐06‐02 10:05:59 UTC	022	34WFT	1.82
S2A_MSIL2A_20200604T100031_N0214_R122_T34WFT_20200604T120105.SAFE	2A	2A	2020‐06‐04 10:00:31 UTC	122	34WFT	1.78
S2B_MSIL2A_20200605T101559_N0214_R065_T34WFT_20200605T135751.SAFE	2B	2A	2020‐06‐05 10:15:59 UTC	065	34WFT	100
S2B_MSIL2A_20200606T095029_N0214_R079_T34WFT_20200606T123908.SAFE	2B	2A	2020‐06‐06 09:50:29 UTC	079	34WFT	55.1
S2A_MSIL2A_20200607T101031_N0214_R022_T34WFT_20200607T124650.SAFE	2A	2A	2020‐06‐07 10:10:31 UTC	022	34WFT	75.7
S2B_MSIL2A_20200609T100029_N0214_R122_T34WFT_20200609T130030.SAFE	2B	2A	2020‐06‐09 10:00:29 UTC	122	34WFT	0.5
S2A_MSIL2A_20200610T102031_N0214_R065_T34WFT_20200610T125245.SAFE	2A	2A	2020‐06‐10 10:20:31 UTC	065	34WFT	100
S2A_MSIL2A_20200611T095041_N0214_R079_T34WFT_20200611T112354.SAFE	2A	2A	2020‐06‐11 09:50:41 UTC	079	34WFT	11.3
S2B_MSIL2A_20200612T100559_N0214_R022_T34WFT_20200612T133209.SAFE	2B	2A	2020‐06‐12 10:05:59 UTC	022	34WFT	0.6
S2A_MSIL2A_20200614T100031_N0214_R122_T34WFT_20200614T123011.SAFE	2A	2A	2020‐06‐14 10:00:31 UTC	122	34WFT	0.52
S2B_MSIL2A_20200615T101559_N0214_R065_T34WFT_20200615T141117.SAFE	2B	2A	2020‐06‐15 10:15:59 UTC	065	34WFT	100
S2B_MSIL2A_20200616T095029_N0214_R079_T34WFT_20200616T123759.SAFE	2B	2A	2020‐06‐16 09:50:29 UTC	079	34WFT	0.9
S2A_MSIL2A_20200617T101031_N0214_R022_T34WFT_20200617T161854.SAFE	2A	2A	2020‐06‐17 10:10:31 UTC	022	34WFT	0.26
S2B_MSIL2A_20200619T100029_N0214_R122_T34WFT_20200619T130948.SAFE	2B	2A	2020‐06‐19 10:00:29 UTC	122	34WFT	38.06
S2A_MSIL2A_20200620T102031_N0214_R065_T34WFT_20200620T110153.SAFE	2A	2A	2020‐06‐20 10:20:31 UTC	065	34WFT	100
S2A_MSIL2A_20200621T095041_N0214_R079_T34WFT_20200621T123605.SAFE	2A	2A	2020‐06‐21 09:50:41 UTC	079	34WFT	0.5
S2B_MSIL2A_20200622T100559_N0214_R022_T34WFT_20200622T134211.SAFE	2B	2A	2020‐06‐22 10:05:59 UTC	022	34WFT	0.23
S2A_MSIL2A_20200624T100031_N0214_R122_T34WFT_20200624T114230.SAFE	2A	2A	2020‐06‐24 10:00:31 UTC	122	34WFT	0.82
S2B_MSIL2A_20200625T101559_N0214_R065_T34WFT_20200625T134347.SAFE	2B	2A	2020‐06‐25 10:15:59 UTC	065	34WFT	100
S2B_MSIL2A_20210515T100029_N0300_R122_T34WFT_20210515T113907.SAFE	2B	2A	2021‐05‐15 10:00:29 UTC	122	34WFT	91.32
S2A_MSIL2A_20210516T102021_N0300_R065_T34WFT_20210516T112130.SAFE	2A	2A	2021‐05‐16 10:20:21 UTC	065	34WFT	100
S2A_MSIL2A_20210517T095031_N0300_R079_T34WFT_20210517T104349.SAFE	2A	2A	2021‐05‐17 09:50:31 UTC	079	34WFT	13.23
S2B_MSIL2A_20210518T100559_N0300_R022_T34WFT_20210518T123536.SAFE	2B	2A	2021‐05‐18 10:05:59 UTC	022	34WFT	100
S2A_MSIL2A_20210520T100031_N0300_R122_T34WFT_20210520T114433.SAFE	2A	2A	2021‐05‐20 10:00:31 UTC	122	34WFT	100
S2B_MSIL2A_20210521T101559_N0300_R065_T34WFT_20210521T131630.SAFE	2B	2A	2021‐05‐21 10:15:59 UTC	065	34WFT	100
S2B_MSIL2A_20210522T095029_N0300_R079_T34WFT_20210522T113428.SAFE	2B	2A	2021‐05‐22 09:50:29 UTC	079	34WFT	100
S2A_MSIL2A_20210523T101031_N0300_R022_T34WFT_20210523T110728.SAFE	2A	2A	2021‐05‐23 10:10:31 UTC	022	34WFT	100
S2B_MSIL2A_20210525T100029_N0300_R122_T34WFT_20210526T150239.SAFE	2B	2A	2021‐05‐25 10:00:29 UTC	122	34WFT	100
S2A_MSIL2A_20210526T102021_N0300_R065_T34WFT_20210526T112505.SAFE	2A	2A	2021‐05‐26 10:20:21 UTC	065	34WFT	100
S2A_MSIL2A_20210527T095031_N0300_R079_T34WFT_20210527T104343.SAFE	2A	2A	2021‐05‐27 09:50:31 UTC	079	34WFT	100
S2B_MSIL2A_20210528T100559_N0300_R022_T34WFT_20210528T130836.SAFE	2B	2A	2021‐05‐28 10:05:59 UTC	022	34WFT	7.35
S2A_MSIL2A_20210530T100031_N0300_R122_T34WFT_20210530T104721.SAFE	2A	2A	2021‐05‐30 10:00:31 UTC	122	34WFT	2.79
S2B_MSIL2A_20210531T101559_N0300_R065_T34WFT_20210531T131117.SAFE	2B	2A	2021‐05‐31 10:15:59 UTC	065	34WFT	100
S2B_MSIL2A_20210601T095029_N0300_R079_T34WFT_20210601T121934.SAFE	2B	2A	2021‐06‐01 09:50:29 UTC	079	34WFT	100
S2A_MSIL2A_20210602T101031_N0300_R022_T34WFT_20210602T150049.SAFE	2A	2A	2021‐06‐02 10:10:31 UTC	022	34WFT	1.45
S2B_MSIL2A_20210604T100029_N0300_R122_T34WFT_20210604T131007.SAFE	2B	2A	2021‐06‐04 10:00:29 UTC	122	34WFT	0.93
S2A_MSIL2A_20210605T102021_N0300_R065_T34WFT_20210605T132908.SAFE	2A	2A	2021‐06‐05 10:20:21 UTC	065	34WFT	100
S2A_MSIL2A_20210606T095031_N0300_R079_T34WFT_20210606T104358.SAFE	2A	2A	2021‐06‐06 09:50:31 UTC	079	34WFT	1.42
S2B_MSIL2A_20210607T100559_N0300_R022_T34WFT_20210607T135736.SAFE	2B	2A	2021‐06‐07 10:05:59 UTC	022	34WFT	2.13
S2A_MSIL2A_20210609T100031_N0300_R122_T34WFT_20210609T105724.SAFE	2A	2A	2021‐06‐09 10:00:31 UTC	122	34WFT	98.91
S2B_MSIL2A_20210610T101559_N0300_R065_T34WFT_20210610T122955.SAFE	2B	2A	2021‐06‐10 10:15:59 UTC	065	34WFT	100
S2B_MSIL2A_20210611T095029_N0300_R079_T34WFT_20210611T122043.SAFE	2B	2A	2021‐06‐11 09:50:29 UTC	079	34WFT	44.75
S2A_MSIL2A_20210612T101031_N0300_R022_T34WFT_20210612T114929.SAFE	2A	2A	2021‐06‐12 10:10:31 UTC	022	34WFT	100
S2B_MSIL2A_20210614T100029_N0300_R122_T34WFT_20210614T112518.SAFE	2B	2A	2021‐06‐14 10:00:29 UTC	122	34WFT	100
S2A_MSIL2A_20210615T102021_N0300_R065_T34WFT_20210615T111718.SAFE	2A	2A	2021‐06‐15 10:20:21 UTC	065	34WFT	100
S2A_MSIL2A_20210616T095031_N0300_R079_T34WFT_20210616T143314.SAFE	2A	2A	2021‐06‐16 09:50:31 UTC	079	34WFT	100
S2B_MSIL2A_20210617T100559_N0300_R022_T34WFT_20210617T122032.SAFE	2B	2A	2021‐06‐17 10:05:59 UTC	022	34WFT	44.07
S2A_MSIL2A_20210619T100031_N0300_R122_T34WFT_20210619T114431.SAFE	2A	2A	2021‐06‐19 10:00:31 UTC	122	34WFT	16.03
S2B_MSIL2A_20210620T101559_N0300_R065_T34WFT_20210620T130659.SAFE	2B	2A	2021‐06‐20 10:15:59 UTC	065	34WFT	100
S2B_MSIL2A_20210621T095029_N0300_R079_T34WFT_20210621T113730.SAFE	2B	2A	2021‐06‐21 09:50:29 UTC	079	34WFT	100
S2A_MSIL2A_20210622T101031_N0300_R022_T34WFT_20210622T110641.SAFE	2A	2A	2021‐06‐22 10:10:31 UTC	022	34WFT	54.02
S2B_MSIL2A_20210624T100029_N0300_R122_T34WFT_20210624T121647.SAFE	2B	2A	2021‐06‐24 10:00:29 UTC	122	34WFT	1.79
S2A_MSIL2A_20210625T102021_N0300_R065_T34WFT_20210625T111856.SAFE	2A	2A	2021‐06‐25 10:20:21 UTC	065	34WFT	100
S2A_MSIL2A_20220515T100031_N0400_R122_T34WFT_20220515T141508.SAFE	2A	2A	2022‐05‐15 10:00:31 UTC	122	34WFT	100
S2B_MSIL2A_20220516T101559_N0400_R065_T34WFT_20220516T130545.SAFE	2B	2A	2022‐05‐16 10:15:59 UTC	065	34WFT	100
S2B_MSIL2A_20220517T095029_N0400_R079_T34WFT_20220517T124459.SAFE	2B	2A	2022‐05‐17 09:50:29 UTC	079	34WFT	100
S2A_MSIL2A_20220518T100601_N0400_R022_T34WFT_20220518T162816.SAFE	2A	2A	2022‐05‐18 10:06:01 UTC	022	34WFT	100
S2B_MSIL2A_20220520T100029_N0400_R122_T34WFT_20220520T115210.SAFE	2B	2A	2022‐05‐20 10:00:29 UTC	122	34WFT	100
S2A_MSIL2A_20220521T101601_N0400_R065_T34WFT_20220521T181716.SAFE	2A	2A	2022‐05‐21 10:16:01 UTC	065	34WFT	100
S2A_MSIL2A_20220522T095041_N0400_R079_T34WFT_20220522T142504.SAFE	2A	2A	2022‐05‐22 09:50:41 UTC	079	34WFT	100
S2B_MSIL2A_20220523T100559_N0400_R022_T34WFT_20220523T115247.SAFE	2B	2A	2022‐05‐23 10:05:59 UTC	022	34WFT	100
S2A_MSIL2A_20220525T100031_N0400_R122_T34WFT_20220525T141308.SAFE	2A	2A	2022‐05‐25 10:00:31 UTC	122	34WFT	100
S2B_MSIL2A_20220526T101559_N0400_R065_T34WFT_20220526T115231.SAFE	2B	2A	2022‐05‐26 10:15:59 UTC	065	34WFT	100
S2B_MSIL2A_20220527T095029_N0400_R079_T34WFT_20220527T115525.SAFE	2B	2A	2022‐05‐27 09:50:29 UTC	079	34WFT	100
S2A_MSIL2A_20220528T100601_N0400_R022_T34WFT_20220528T162819.SAFE	2A	2A	2022‐05‐28 10:06:01 UTC	022	34WFT	100
S2B_MSIL2A_20220530T100029_N0400_R122_T34WFT_20220530T115104.SAFE	2B	2A	2022‐05‐30 10:00:29 UTC	122	34WFT	100
S2A_MSIL2A_20220531T101611_N0400_R065_T34WFT_20220531T163910.SAFE	2A	2A	2022‐05‐31 10:16:11 UTC	065	34WFT	100
S2A_MSIL2A_20220601T095041_N0400_R079_T34WFT_20220601T142611.SAFE	2A	2A	2022‐06‐01 09:50:41 UTC	079	34WFT	100
S2B_MSIL2A_20220602T100559_N0400_R022_T34WFT_20220602T181336.SAFE	2B	2A	2022‐06‐02 10:05:59 UTC	022	34WFT	100
S2A_MSIL2A_20220604T100031_N0400_R122_T34WFT_20220604T141210.SAFE	2A	2A	2022‐06‐04 10:00:31 UTC	122	34WFT	100
S2B_MSIL2A_20220605T101559_N0400_R065_T34WFT_20220605T120530.SAFE	2B	2A	2022‐06‐05 10:15:59 UTC	065	34WFT	100
S2B_MSIL2A_20220606T095029_N0400_R079_T34WFT_20220606T115947.SAFE	2B	2A	2022‐06‐06 09:50:29 UTC	079	34WFT	100
S2A_MSIL2A_20220607T100601_N0400_R022_T34WFT_20220607T162619.SAFE	2A	2A	2022‐06‐07 10:06:01 UTC	022	34WFT	100
S2B_MSIL2A_20220609T100029_N0400_R122_T34WFT_20220614T114726.SAFE	2B	2A	2022‐06‐09 10:00:29 UTC	122	34WFT	100
S2A_MSIL2A_20220610T101611_N0400_R065_T34WFT_20220610T171812.SAFE	2A	2A	2022‐06‐10 10:16:11 UTC	065	34WFT	100
S2A_MSIL2A_20220611T095041_N0400_R079_T34WFT_20220611T143810.SAFE	2A	2A	2022‐06‐11 09:50:41 UTC	079	34WFT	100
S2B_MSIL2A_20220612T100559_N0400_R022_T34WFT_20220612T115926.SAFE	2B	2A	2022‐06‐12 10:05:59 UTC	022	34WFT	100
S2A_MSIL2A_20220614T100041_N0400_R122_T34WFT_20220614T143516.SAFE	2A	2A	2022‐06‐14 10:00:41 UTC	122	34WFT	100
S2B_MSIL2A_20220615T101559_N0400_R065_T34WFT_20220615T113847.SAFE	2B	2A	2022‐06‐15 10:15:59 UTC	065	34WFT	100
S2B_MSIL2A_20220616T095039_N0400_R079_T34WFT_20220616T124856.SAFE	2B	2A	2022‐06‐16 09:50:39 UTC	079	34WFT	100
S2A_MSIL2A_20220617T100611_N0400_R022_T34WFT_20220617T162624.SAFE	2A	2A	2022‐06‐17 10:06:11 UTC	022	34WFT	100
S2B_MSIL2A_20220619T100029_N0400_R122_T34WFT_20220619T114329.SAFE	2B	2A	2022‐06‐19 10:00:29 UTC	122	34WFT	100
S2A_MSIL2A_20220620T102041_N0400_R065_T34WFT_20220620T162319.SAFE	2A	2A	2022‐06‐20 10:20:41 UTC	065	34WFT	100
S2A_MSIL2A_20220621T095041_N0400_R079_T34WFT_20220621T142717.SAFE	2A	2A	2022‐06‐21 09:50:41 UTC	079	34WFT	100
S2B_MSIL2A_20220622T100559_N0400_R022_T34WFT_20220622T115236.SAFE	2B	2A	2022‐06‐22 10:05:59 UTC	022	34WFT	100
S2A_MSIL2A_20220624T100041_N0400_R122_T34WFT_20220624T143914.SAFE	2A	2A	2022‐06‐24 10:00:41 UTC	122	34WFT	100
S2B_MSIL2A_20220625T101559_N0400_R065_T34WFT_20220625T114347.SAFE	2B	2A	2022‐06‐25 10:15:59 UTC	065	34WFT	100

*Note:* NDVI images downloaded for the field area for the years 2018–2022, between May 15th and June 25th, indicating the percentage of cloud cover.

**TABLE A2:** SAR image metadata.

Granule name	Platform	Orbit	Path number	Frame number	Acquisition date	Processing level	URL
S1A_IW_GRDH_1SDV_20220624T155816_20220624T155841_043807_053AD2_0F95	Sentinel‐1A	43,807	160	208	2022‐06‐24T15:58:41.000000	GRD_HD	https://datapool.asf.alaska.edu/GRD_HD/SA/S1A_IW_GRDH_1SDV_20220624T155816_20220624T155841_043807_053AD2_0F95.zip
S1A_IW_GRDH_1SDV_20220619T155015_20220619T155040_043734_0538A8_1C9C	Sentinel‐1A	43,734	87	211	2022‐06‐19T15:50:40.000000	GRD_HD	https://datapool.asf.alaska.edu/GRD_HD/SA/S1A_IW_GRDH_1SDV_20220619T155015_20220619T155040_043734_0538A8_1C9C.zip
S1A_IW_GRDH_1SDV_20220614T154200_20220614T154225_043661_05366D_E85F	Sentinel‐1A	43,661	14	211	2022‐06‐14T15:42:25.000000	GRD_HD	https://datapool.asf.alaska.edu/GRD_HD/SA/S1A_IW_GRDH_1SDV_20220614T154200_20220614T154225_043661_05366D_E85F.zip
S1A_IW_GRDH_1SDV_20220612T155815_20220612T155840_043632_053593_1D06	Sentinel‐1A	43,632	160	208	2022‐06‐12T15:58:40.000000	GRD_HD	https://datapool.asf.alaska.edu/GRD_HD/SA/S1A_IW_GRDH_1SDV_20220612T155815_20220612T155840_043632_053593_1D06.zip
S1A_IW_GRDH_1SDV_20220607T155014_20220607T155039_043559_053375_3608	Sentinel‐1A	43,559	87	211	2022‐06‐07T15:50:39.000000	GRD_HD	https://datapool.asf.alaska.edu/GRD_HD/SA/S1A_IW_GRDH_1SDV_20220607T155014_20220607T155039_043559_053375_3608.zip
S1A_IW_GRDH_1SDV_20220602T154159_20220602T154224_043486_053134_B8A2	Sentinel‐1A	43,486	14	211	2022‐06‐02T15:42:24.000000	GRD_HD	https://datapool.asf.alaska.edu/GRD_HD/SA/S1A_IW_GRDH_1SDV_20220602T154159_20220602T154224_043486_053134_B8A2.zip
S1A_IW_GRDH_1SDV_20220531T155814_20220531T155839_043457_053067_FB8C	Sentinel‐1A	43,457	160	208	2022‐05‐31T15:58:39.000000	GRD_HD	https://datapool.asf.alaska.edu/GRD_HD/SA/S1A_IW_GRDH_1SDV_20220531T155814_20220531T155839_043457_053067_FB8C.zip
S1A_IW_GRDH_1SDV_20220526T155000_20220526T155025_043384_052E44_73A8	Sentinel‐1A	43,384	87	208	2022‐05‐26T15:50:25.000000	GRD_HD	https://datapool.asf.alaska.edu/GRD_HD/SA/S1A_IW_GRDH_1SDV_20220526T155000_20220526T155025_043384_052E44_73A8.zip
S1A_IW_GRDH_1SDV_20220521T154158_20220521T154223_043311_052C0D_CB3A	Sentinel‐1A	43,311	14	211	2022‐05‐21T15:42:23.000000	GRD_HD	https://datapool.asf.alaska.edu/GRD_HD/SA/S1A_IW_GRDH_1SDV_20220521T154158_20220521T154223_043311_052C0D_CB3A.zip
S1A_IW_GRDH_1SDV_20220519T155813_20220519T155838_043282_052B32_96F9	Sentinel‐1A	43,282	160	208	2022‐05‐19T15:58:38.000000	GRD_HD	https://datapool.asf.alaska.edu/GRD_HD/SA/S1A_IW_GRDH_1SDV_20220519T155813_20220519T155838_043282_052B32_96F9.zip
S1A_IW_GRDH_1SDV_20210624T155009_20210624T155034_038484_048A94_5529	Sentinel‐1A	38,484	87	211	2021‐06‐24T15:50:34.000000	GRD_HD	https://datapool.asf.alaska.edu/GRD_HD/SA/S1A_IW_GRDH_1SDV_20210624T155009_20210624T155034_038484_048A94_5529.zip
S1A_IW_GRDH_1SDV_20210619T154154_20210619T154219_038411_048859_DF0F	Sentinel‐1A	38,411	14	211	2021‐06‐19T15:42:19.000000	GRD_HD	https://datapool.asf.alaska.edu/GRD_HD/SA/S1A_IW_GRDH_1SDV_20210619T154154_20210619T154219_038411_048859_DF0F.zip
S1A_IW_GRDH_1SDV_20210617T155809_20210617T155834_038382_048784_96B2	Sentinel‐1A	38,382	160	208	2021‐06‐17T15:58:34.000000	GRD_HD	https://datapool.asf.alaska.edu/GRD_HD/SA/S1A_IW_GRDH_1SDV_20210617T155809_20210617T155834_038382_048784_96B2.zip
S1A_IW_GRDH_1SDV_20210612T155008_20210612T155033_038309_04855A_5F95	Sentinel‐1A	38,309	87	211	2021‐06‐12T15:50:33.000000	GRD_HD	https://datapool.asf.alaska.edu/GRD_HD/SA/S1A_IW_GRDH_1SDV_20210612T155008_20210612T155033_038309_04855A_5F95.zip
S1A_IW_GRDH_1SDV_20210607T154153_20210607T154218_038236_048325_5EB6	Sentinel‐1A	38,236	14	211	2021‐06‐07T15:42:18.000000	GRD_HD	https://datapool.asf.alaska.edu/GRD_HD/SA/S1A_IW_GRDH_1SDV_20210607T154153_20210607T154218_038236_048325_5EB6.zip
S1A_IW_GRDH_1SDV_20210605T155809_20210605T155834_038207_048252_5365	Sentinel‐1A	38,207	160	208	2021‐06‐05T15:58:34.000000	GRD_HD	https://datapool.asf.alaska.edu/GRD_HD/SA/S1A_IW_GRDH_1SDV_20210605T155809_20210605T155834_038207_048252_5365.zip
S1A_IW_GRDH_1SDV_20210531T155008_20210531T155033_038134_04802D_A079	Sentinel‐1A	38,134	87	211	2021‐05‐31T15:50:33.000000	GRD_HD	https://datapool.asf.alaska.edu/GRD_HD/SA/S1A_IW_GRDH_1SDV_20210531T155008_20210531T155033_038134_04802D_A079.zip
S1A_IW_GRDH_1SDV_20210526T154153_20210526T154218_038061_047DF2_4544	Sentinel‐1A	38,061	14	211	2021‐05‐26T15:42:18.000000	GRD_HD	https://datapool.asf.alaska.edu/GRD_HD/SA/S1A_IW_GRDH_1SDV_20210526T154153_20210526T154218_038061_047DF2_4544.zip
S1A_IW_GRDH_1SDV_20210524T155808_20210524T155833_038032_047D1C_3E7A	Sentinel‐1A	38,032	160	208	2021‐05‐24T15:58:33.000000	GRD_HD	https://datapool.asf.alaska.edu/GRD_HD/SA/S1A_IW_GRDH_1SDV_20210524T155808_20210524T155833_038032_047D1C_3E7A.zip
S1A_IW_GRDH_1SDV_20210519T155007_20210519T155032_037959_047AEC_30E3	Sentinel‐1A	37,959	87	211	2021‐05‐19T15:50:32.000000	GRD_HD	https://datapool.asf.alaska.edu/GRD_HD/SA/S1A_IW_GRDH_1SDV_20210519T155007_20210519T155032_037959_047AEC_30E3.zip
S1A_IW_GRDH_1SDV_20200624T154148_20200624T154213_033161_03D770_C9FA	Sentinel‐1A	33,161	14	211	2020‐06‐24T15:42:13.000000	GRD_HD	https://datapool.asf.alaska.edu/GRD_HD/SA/S1A_IW_GRDH_1SDV_20200624T154148_20200624T154213_033161_03D770_C9FA.zip
S1A_IW_GRDH_1SDV_20200622T155804_20200622T155829_033132_03D695_9B9B	Sentinel‐1A	33,132	160	208	2020‐06‐22T15:58:29.000000	GRD_HD	https://datapool.asf.alaska.edu/GRD_HD/SA/S1A_IW_GRDH_1SDV_20200622T155804_20200622T155829_033132_03D695_9B9B.zip
S1A_IW_GRDH_1SDV_20200617T155003_20200617T155028_033059_03D45D_3853	Sentinel‐1A	33,059	87	211	2020‐06‐17T15:50:28.000000	GRD_HD	https://datapool.asf.alaska.edu/GRD_HD/SA/S1A_IW_GRDH_1SDV_20200617T155003_20200617T155028_033059_03D45D_3853.zip
S1A_IW_GRDH_1SDV_20200612T154148_20200612T154213_032986_03D21B_D67E	Sentinel‐1A	32,986	14	211	2020‐06‐12T15:42:13.000000	GRD_HD	https://datapool.asf.alaska.edu/GRD_HD/SA/S1A_IW_GRDH_1SDV_20200612T154148_20200612T154213_032986_03D21B_D67E.zip
S1A_IW_GRDH_1SDV_20200610T155803_20200610T155828_032957_03D144_AA0D	Sentinel‐1A	32,957	160	208	2020‐06‐10T15:58:28.000000	GRD_HD	https://datapool.asf.alaska.edu/GRD_HD/SA/S1A_IW_GRDH_1SDV_20200610T155803_20200610T155828_032957_03D144_AA0D.zip
S1A_IW_GRDH_1SDV_20200605T155002_20200605T155027_032884_03CF19_23F7	Sentinel‐1A	32,884	87	211	2020‐06‐05T15:50:27.000000	GRD_HD	https://datapool.asf.alaska.edu/GRD_HD/SA/S1A_IW_GRDH_1SDV_20200605T155002_20200605T155027_032884_03CF19_23F7.zip
S1A_IW_GRDH_1SDV_20200531T154147_20200531T154212_032811_03CCEF_5006	Sentinel‐1A	32,811	14	211	2020‐05‐31T15:42:12.000000	GRD_HD	https://datapool.asf.alaska.edu/GRD_HD/SA/S1A_IW_GRDH_1SDV_20200531T154147_20200531T154212_032811_03CCEF_5006.zip
S1A_IW_GRDH_1SDV_20200529T155802_20200529T155827_032782_03CC1B_4683	Sentinel‐1A	32,782	160	208	2020‐05‐29T15:58:27.000000	GRD_HD	https://datapool.asf.alaska.edu/GRD_HD/SA/S1A_IW_GRDH_1SDV_20200529T155802_20200529T155827_032782_03CC1B_4683.zip
S1A_IW_GRDH_1SDV_20200524T155001_20200524T155026_032709_03C9EC_B197	Sentinel‐1A	32,709	87	211	2020‐05‐24T15:50:26.000000	GRD_HD	https://datapool.asf.alaska.edu/GRD_HD/SA/S1A_IW_GRDH_1SDV_20200524T155001_20200524T155026_032709_03C9EC_B197.zip
S1A_IW_GRDH_1SDV_20200519T154146_20200519T154211_032636_03C7A7_0780	Sentinel‐1A	32,636	14	211	2020‐05‐19T15:42:11.000000	GRD_HD	https://datapool.asf.alaska.edu/GRD_HD/SA/S1A_IW_GRDH_1SDV_20200519T154146_20200519T154211_032636_03C7A7_0780.zip
S1A_IW_GRDH_1SDV_20200517T155802_20200517T155827_032607_03C6D3_03D7	Sentinel‐1A	32,607	160	208	2020‐05‐17T15:58:27.000000	GRD_HD	https://datapool.asf.alaska.edu/GRD_HD/SA/S1A_IW_GRDH_1SDV_20200517T155802_20200517T155827_032607_03C6D3_03D7.zip
S1A_IW_GRDH_1SDV_20190623T154956_20190623T155021_027809_0323A7_309C	Sentinel‐1A	27,809	87	211	2019‐06‐23T15:50:21.000000	GRD_HD	https://datapool.asf.alaska.edu/GRD_HD/SA/S1A_IW_GRDH_1SDV_20190623T154956_20190623T155021_027809_0323A7_309C.zip
S1A_IW_GRDH_1SDV_20190618T154141_20190618T154206_027736_032175_E0E6	Sentinel‐1A	27,736	14	211	2019‐06‐18T15:42:06.000000	GRD_HD	https://datapool.asf.alaska.edu/GRD_HD/SA/S1A_IW_GRDH_1SDV_20190618T154141_20190618T154206_027736_032175_E0E6.zip
S1A_IW_GRDH_1SDV_20190616T155756_20190616T155821_027707_0320A4_88DF	Sentinel‐1A	27,707	160	208	2019‐06‐16T15:58:21.000000	GRD_HD	https://datapool.asf.alaska.edu/GRD_HD/SA/S1A_IW_GRDH_1SDV_20190616T155756_20190616T155821_027707_0320A4_88DF.zip
S1A_IW_GRDH_1SDV_20190611T154955_20190611T155020_027634_031E6F_C9C4	Sentinel‐1A	27,634	87	211	2019‐06‐11T15:50:20.000000	GRD_HD	https://datapool.asf.alaska.edu/GRD_HD/SA/S1A_IW_GRDH_1SDV_20190611T154955_20190611T155020_027634_031E6F_C9C4.zip
S1A_IW_GRDH_1SDV_20190606T154141_20190606T154206_027561_031C2F_E648	Sentinel‐1A	27,561	14	211	2019‐06‐06T15:42:06.000000	GRD_HD	https://datapool.asf.alaska.edu/GRD_HD/SA/S1A_IW_GRDH_1SDV_20190606T154141_20190606T154206_027561_031C2F_E648.zip
S1A_IW_GRDH_1SDV_20190604T155756_20190604T155821_027532_031B5B_13C2	Sentinel‐1A	27,532	160	208	2019‐06‐04T15:58:21.000000	GRD_HD	https://datapool.asf.alaska.edu/GRD_HD/SA/S1A_IW_GRDH_1SDV_20190604T155756_20190604T155821_027532_031B5B_13C2.zip
S1A_IW_GRDH_1SDV_20190530T154955_20190530T155020_027459_031914_DFDA	Sentinel‐1A	27,459	87	211	2019‐05‐30T15:50:20.000000	GRD_HD	https://datapool.asf.alaska.edu/GRD_HD/SA/S1A_IW_GRDH_1SDV_20190530T154955_20190530T155020_027459_031914_DFDA.zip
S1A_IW_GRDH_1SDV_20190525T154140_20190525T154205_027386_0316C5_BB1F	Sentinel‐1A	27,386	14	211	2019‐05‐25T15:42:05.000000	GRD_HD	https://datapool.asf.alaska.edu/GRD_HD/SA/S1A_IW_GRDH_1SDV_20190525T154140_20190525T154205_027386_0316C5_BB1F.zip
S1A_IW_GRDH_1SDV_20190523T155755_20190523T155820_027357_0315EB_3479	Sentinel‐1A	27,357	160	208	2019‐05‐23T15:58:20.000000	GRD_HD	https://datapool.asf.alaska.edu/GRD_HD/SA/S1A_IW_GRDH_1SDV_20190523T155755_20190523T155820_027357_0315EB_3479.zip
S1A_IW_GRDH_1SDV_20190518T154954_20190518T155019_027284_03139F_6444	Sentinel‐1A	27,284	87	211	2019‐05‐18T15:50:19.000000	GRD_HD	https://datapool.asf.alaska.edu/GRD_HD/SA/S1A_IW_GRDH_1SDV_20190518T154954_20190518T155019_027284_03139F_6444.zip
S1A_IW_GRDH_1SDV_20180623T154136_20180623T154201_022486_026F73_5825	Sentinel‐1A	22,486	14	211	2018‐06‐23T15:42:01.000000	GRD_HD	https://datapool.asf.alaska.edu/GRD_HD/SA/S1A_IW_GRDH_1SDV_20180623T154136_20180623T154201_022486_026F73_5825.zip
S1A_IW_GRDH_1SDV_20180621T155751_20180621T155816_022457_026EA0_F922	Sentinel‐1A	22,457	160	208	2018‐06‐21T15:58:16.000000	GRD_HD	https://datapool.asf.alaska.edu/GRD_HD/SA/S1A_IW_GRDH_1SDV_20180621T155751_20180621T155816_022457_026EA0_F922.zip
S1A_IW_GRDH_1SDV_20180616T154950_20180616T155015_022384_026C74_0188	Sentinel‐1A	22,384	87	211	2018‐06‐16T15:50:15.000000	GRD_HD	https://datapool.asf.alaska.edu/GRD_HD/SA/S1A_IW_GRDH_1SDV_20180616T154950_20180616T155015_022384_026C74_0188.zip
S1A_IW_GRDH_1SDV_20180611T154135_20180611T154200_022311_026A34_CB67	Sentinel‐1A	22,311	14	211	2018‐06‐11T15:42:00.000000	GRD_HD	https://datapool.asf.alaska.edu/GRD_HD/SA/S1A_IW_GRDH_1SDV_20180611T154135_20180611T154200_022311_026A34_CB67.zip
S1A_IW_GRDH_1SDV_20180609T155750_20180609T155815_022282_02694E_6089	Sentinel‐1A	22,282	160	208	2018‐06‐09T15:58:15.000000	GRD_HD	https://datapool.asf.alaska.edu/GRD_HD/SA/S1A_IW_GRDH_1SDV_20180609T155750_20180609T155815_022282_02694E_6089.zip
S1A_IW_GRDH_1SDV_20180604T154949_20180604T155014_022209_02670F_5030	Sentinel‐1A	22,209	87	211	2018‐06‐04T15:50:14.000000	GRD_HD	https://datapool.asf.alaska.edu/GRD_HD/SA/S1A_IW_GRDH_1SDV_20180604T154949_20180604T155014_022209_02670F_5030.zip
S1A_IW_GRDH_1SDV_20180530T154134_20180530T154159_022136_0264BF_353B	Sentinel‐1A	22,136	14	211	2018‐05‐30T15:41:59.000000	GRD_HD	https://datapool.asf.alaska.edu/GRD_HD/SA/S1A_IW_GRDH_1SDV_20180530T154134_20180530T154159_022136_0264BF_353B.zip
S1A_IW_GRDH_1SDV_20180528T155749_20180528T155814_022107_0263D7_9A52	Sentinel‐1A	22,107	160	208	2018‐05‐28T15:58:14.000000	GRD_HD	https://datapool.asf.alaska.edu/GRD_HD/SA/S1A_IW_GRDH_1SDV_20180528T155749_20180528T155814_022107_0263D7_9A52.zip
S1A_IW_GRDH_1SDV_20180523T154949_20180523T155014_022034_026183_7267	Sentinel‐1A	22,034	87	211	2018‐05‐23T15:50:14.000000	GRD_HD	https://datapool.asf.alaska.edu/GRD_HD/SA/S1A_IW_GRDH_1SDV_20180523T154949_20180523T155014_022034_026183_7267.zip
S1A_IW_GRDH_1SDV_20180518T154133_20180518T154158_021961_025F1E_159F	Sentinel‐1A	21,961	14	211	2018‐05‐18T15:41:58.000000	GRD_HD	https://datapool.asf.alaska.edu/GRD_HD/SA/S1A_IW_GRDH_1SDV_20180518T154133_20180518T154158_021961_025F1E_159F.zip
S1A_IW_GRDH_1SDV_20180516T155749_20180516T155814_021932_025E39_41FB	Sentinel‐1A	21,932	160	208	2018‐05‐16T15:58:14.000000	GRD_HD	https://datapool.asf.alaska.edu/GRD_HD/SA/S1A_IW_GRDH_1SDV_20180516T155749_20180516T155814_021932_025E39_41FB.zip

*Note:* SAR images downloaded for the field area for the years 2018 to 2022, between May 15 and June 25.
